# Complex PTSD and borderline personality disorder

**DOI:** 10.1186/s40479-021-00155-9

**Published:** 2021-05-06

**Authors:** Julian D. Ford, Christine A. Courtois

**Affiliations:** 1 University of Connecticut Health Center MC1410, 263 Farmington Avenue, Farmington, CT 06030-1410 USA; 2 Independent Pactice, Retired, P.O. Box 1326, Bethany Beach, DE 19930 USA

**Keywords:** Borderline personality disorder, Complex PTSD, Emotion dysregulation

## Abstract

**Background:**

This article builds on a previous review (Ford and Courtois, Borderline Personal Disord Emot Dysregul 1:9, 2014) which concluded that complex posttraumatic stress disorder (cPTSD) could not be conceptualized as a sub-type of either PTSD or BPD. Recent research is reviewed that extends and clarifies the still nascent understanding of the relationship between cPTSD and BPD.

**Main body:**

The circumscribed formulation of adult cPTSD that has been developed, validated, and included in the 11th Edition of the *International Classification of Diseases* has spurred research aimed at differentiating cPTSD and BPD both descriptively and empirically. A newly validated Developmental Trauma Disorder (DTD) syndrome for children and adolescents provides a basis for systematic research on the developmental course and origins of adult cPTSD and BPD. This review summarizes recent empirical findings regarding BPD, PTSD, and cPTSD in terms of: (1) prevalence and comorbidity; (2) clinical phenomenology; (3) traumatic antecedents; (4) psychobiology; (5) emotion dysregulation; (6) dissociation; and (7) empirically supported approaches to clinical assessment and psychotherapeutic treatment.

**Conclusion:**

The evidence suggests that PTSD, cPTSD, and BPD are potentially comorbid but distinct syndromes. A hypothesis is advanced to stimulate scientific research and clinical innovation defining and differentiating the disorders, positing that they may represent a continuum paralleling the classic conceptualization of the stress response, with dissociation potentially involved in each disorder.

## Background

Four decades after complex posttraumatic stress disorder (cPTSD) was first defined and proposed as an alternative diagnosis to borderline personality disorder (BPD) for conceptualizing and treating the symptoms of adults who had suffered prolonged and severe interpersonal trauma, [70], the validity and utility of cPTSD continues to be debated by mental health researchers [39, 41]; however, it is accepted by many clinical practitioners who view it as a less stigmatizing and possibly more accurate diagnosis than BPD [99]. Seven years after a foundational review on cPTSD and BPD [43], the relationship of cPTSD to BPD remains an open question.

cPTSD was originally defined as a disorder of extreme stress not otherwise specified (DESNOS) following traumatic victimization with symptoms in seven domains: emotion dysregulation, altered schemas of self, altered relationships, trauma-related sustaining beliefs (morality, spirituality), somatization, and altered perceptions of perpetrators [142]. Although research with adults [12] and children [25] accumulated over the next two decades, many of the initially proposed symptoms of cPTSD were not included in a separate diagnosis) in the Fifth Edition of the *Diagnostic and Statistical Manual of Mental Disorders* (*DSM-5* [3];). Instead, the *DSM-5* opted for a broader conceptualization of PTSD with some symptoms similar to those in cPTSD.

However, empirical evidence showing that the core features of cPTSD are distinct clinically and conceptually from PTSD has resulted in a revised formulation of cPTSD [18, 20, 80] that has been adopted as a diagnosis in the 11th Revision of the World Health Organization’s *International Classification of Diseases*. Based on confirmatory factor analysis studies with adults [12], the *ICD-*11 diagnosis of cPTSD is defined operationally as requiring one of the two symptoms from each of three *DSM-IV* criteria for PTSD (i.e., intrusive re-experiencing, avoidance of trauma reminders, hyperarousal) and one of the two symptoms from each of three domains of Disturbances of Self-Organization (DSO). The cPTSD/DSO symptoms represent post-traumatic: (1) emotional numbing and dysregulation, (2) self-perceptions as a failure or worthless, and (3) emotional detachment in relationships.

These refinements in the conceptualization and measurement of cPTSD have spurred new research on the relationship of PTSD, cPTSD/DSO and BPD in adulthood, including their traumatic antecedents in childhood and across developmental epochs. Therefore, this review summarizes recent empirical findings regarding BPD, PTSD, and cPTSD in terms of: (1) prevalence and comorbidity; (2) clinical phenomenology; (3) traumatic antecedents; (4) psychobiology; (5) emotion dysregulation; (6) dissociation; and (7) empirically supported approaches to clinical assessment and psychotherapeutic treatment.

## Main text

### Prevalence and comorbidity of BPD, PTSD, and cPTSD

The estimated prevalence of BPD in the adult general population is 0.7–3.5%, and 9–18% among adults in mental health treatment [31]. PTSD prevalence estimates worldwide among adults in the general population are 3.0–4.4% [90, 162], with a wider range of 2–39% in primary care samples [58]. Prevalence estimates for cPTSD in adult non-clinical community populations based on the *ICD-11* definition are 0.5–7.7% [6, 20, 74, 76, 78, 113] and 36% among adults in mental health treatment [117].

PTSD and BPD often occur comorbidly. In nationally representative samples in the United States, 25–30% of adults meeting criteria for either PTSD or BPD also met criteria for the other disorder, and 30–70% of adults diagnosed with BPD had an episode of PTSD at some point in their lifetime [50]. A 10-year follow-up of adults diagnosed with BPD found that most (85%) who had been initially diagnosed with PTSD were in remission from PTSD but continued to meet criteria for BPD [192]. Of note, almost half of the remitted PTSD cases experienced a recurrence and those with childhood histories of sexual abuse were least likely to remit. More than one in four of the BPD cases had a new diagnosis of PTSD over the 10-year period, most often following a sexual assault [192].

The comorbidity of cPTSD and BPD was reported for the DESNOS version of cPTSD in a Dutch inpatient psychiatric sample [180]: BPD was comorbid in 79% of cPTSD cases and cPTSD was comorbid in 40.5% of BPD cases. In a sample of Danish adults in outpatient psychiatric treatment [117] in which *ICD-11* cPTSD was more prevalent (36%) than BPD (8%) or PTSD (8%), and cPTSD was comorbid in 44% of the BPD cases, while BPD was comorbid in only 10% of the cPTSD cases and 8% of the PTSD cases. Similarly, in an outpatient treatment-seeking sample of women with histories of childhood abuse, *ICD-11* cPTSD was comorbid with approximately half of cases diagnosed with BPD, while BPD was comorbid with only approximately 8% of cases diagnosed with cPTSD [19].

#### Summary

Although the greater severity of psychopathology in inpatient samples may explain the higher comorbidity estimates than in the outpatient samples, across these studies the findings were consistent in indicating that *BPD occurs more often when cPTSD is diagnosed than cPTSD occurs when BPD is diagnosed*. Recent studies that have investigated how the symptoms of the two disorders co-occur will be discussed in the next section on clinical phenomenology, to better understand their distinct features and the symptoms that contribute to their comorbidity. More precise prevalence and comorbidity estimates are needed using the standardized cPTSD assessment developed for the *ICD-11* [19], as well as with a range of trauma-exposed populations globally.

### Clinical phenomenology of BPD, PTSD, and cPTSD

BPD and PTSD are distinct regarding the precise qualitative definitions of their diagnostic features but have been found to have substantial potential overlap in their symptom criteria. In earlier versions of the *DSM* and the *ICD* (i.e., *DSM*-*IV* and *ICD-10*, PTSD symptoms (i.e., dissociative amnesia and flashbacks; emotional numbing; anger) are similar to BPD features of transient dissociation, chronic emptiness, and intense anger. Moreover, the revised PTSD criteria in the *DSM*-5 [3] include new symptoms of pervasive negative changes in cognition, mood, and behavior that parallel other BPD criteria (i.e., identity disturbance, interpersonal mistrust, dysphoric affective instability, impulsivity, self-harm) [148].

In terms of cPTSD, the three DSO domains (i.e., emotion dysregulation, negative self-perception, impaired relationships) also parallel BPD features. However, the specific DSO symptoms are relatively distinct from BPD symptoms. In cPTSD/DSO, emotion dysregulation involves chronic difficulty in self-calming when distressed and chronic emotional numbing, but not the emotional lability, extreme uncontrolled anger, and profound emotional dyscontrol found in BPD. Negative self-perceptions in cPTSD/DSO tend to center on a stable chronic sense of guilt, shame, and worthlessness, in contrast to the more unstable and fragmented sense of self in BPD. Although both cPTSD and BPD involve severe relational problems, this involves intense volatile relational hostility and alternating enmeshment and disengagement to avoid real or imagined abandonment in BPD. In cPTSD/DSO, relational dysregulation instead is characterized by avoidance and detachment based a fear of closeness [18].

Consistent with this analysis, person-centered research studies have provided evidence that BPD and cPTSD constitute somewhat distinct but often overlapping symptom profiles that also often overlap with PTSD symptoms. In a sample of outpatient treatment-seeking women with childhood abuse histories [19], three sub-groups were identified that were characterized by: (1) PTSD with minimal DSO and BPD symptoms except for interpersonal detachment; (2) cPTSD with both PTSD and DSO symptoms and minimal BPD symptoms except emotional emptiness; and (3) BPD plus all PTSD and DSO symptoms except for avoidance. Terror of abandonment, unstable sense of self and relationships, and impulsiveness best distinguished the “BPD” versus cPTSD sub-groups, although dissociation, temper outbursts, self-harm, and mood instability also were twice as likely to occur in the BPD class as in the cPTSD class. However, when BPD symptoms were endorsed they were consistently accompanied by PTSD and cPTSD/DSO symptoms. Thus rather than occurring separately from PTSD or cPTSD, in this trauma-exposed sample BPD symptoms appeared to represent a high severity sub-type of cPTSD, similar to prior results for PTSD and cPTSD [190]. However, the absence of non-trauma-exposed individuals in this sample may have limited the opportunity for BPD to emerge as a syndrome distinct from cPTSD.

In a sample of adults in outpatient psychiatric treatment in Great Britain, three classes in some ways similar but in other ways different from those found by Cloitre et al. [19] were identified ([83], 1) a PTSD class whose members had a high (70–90%) probability of endorsing PTSD symptoms and a low (0–50%) probability of all DSO and BPD symptoms; (2) a cPTSD class characterized by a high (> 90%) probability of PTSD and DSO symptoms, and also the BPD symptom of unstable sense of self; and, (3) a BPD/cPTSD class characterized by a high (> 80%) probability of all PTSD, DSO, and BPD symptoms;. The two cPTSD classes were more likely than the PTSD class to report histories of emotional, sexual, and physical abuse and emotional and physical neglect, and the comorbid BPD/cPTSD class was more likely to report histories of emotional abuse and physical neglect than the cPTSD classes. The PTSD class, on the other hand, was more likely than the BPD/cPTSD class to report experiencing a non-interpersonal traumatic event in adulthood, and specifically in the past 6 months. Although work-related impairment was comparable for the DSO, cPTSD, and BPD/cPTSD classes, members of the cPTSD and DSO classes reported more impairment in household, leisure, and relationship domains than members of the PTSD class, and members of the cPTSD/BPD reported the most severe impairment in the relationship domain [84]. Thus, BPD symptoms were notable primarily among psychiatric outpatients who also reported both extensive PTSD and cPTSD/DSO symptoms, while PTSD and DSO symptoms also occurred apart from BPD symptoms (except for unstable identity) both in combination and separate from one another.

In a general population sample of adults from the US who disclosed a history of sexual trauma, half had minimal BPD symptoms (i.e., 0–20% endorsement) and comprised two distinct sub-groups, one consistent with PTSD (i.e., 33% of the sample, who mainly reported PTSD symptoms and one DSO symptom—difficulty in self-calming when distressed) and one consistent with cPTSD (i.e., 18% of the sample, who often [75–100%] endorsed all PTSD symptoms, but also the emotion and relational dysregulation symptoms of DSO) [52]. Two other sub-groups were characterized by higher (20–85%) levels of endorsement of BPD symptoms: a comorbid BPD/PTSD class (12.5% of the sample, who often endorsed all PTSD symptoms and also the DSO symptom of difficulty in self-calming when distressed), and a comorbid BPD/cPTSD class (17% of the sample, who often endorsed all PTSD and DSO symptoms). A fifth sub-group’s members infrequently endorsed any symptoms (20%). The comorbid BPD/cPTSD sub-group members were more likely to report a history of childhood physical, sexual, and emotional abuse and neglect than the PTSD or low symptom sub-group members. On the other hand, cPTSD sub-group members were more likely to report only one form of childhood adversity (i.e., emotional neglect) than the PTSD or low symptom sub-group members. In this sexual victimization, sample, BPD symptoms again occurred primarily in combination either with all of the PTSD symptoms or with all of the cPTSD (PTSD and DSO) symptoms, and the comorbid BPD/cPTSD cohort was characterized by the most extensive childhood poly-victimization.

With another trauma-exposed non-clinical population sample from the United Kingdom, structural equation modelling identified more clearly distinct PTSD, DSO, and BPD latent variables [73]. Fear-related intrusive memories, avoidance, and hypervigilance were prominent in a PTSD latent variable. A DSO latent variable was characterized by symptoms of emotional numbing and emptiness, self-perception as damaged, and relational detachment. Emotional reactivity, impulsivity, emptiness, terror of abandonment, and a fragmented and unstable sense of self were prominent in a BPD latent variable. Although PTSD, DSO, and BPD were clearly distinct as latent variables, contrary to expectations, all three were associated with self-reported histories of childhood interpersonal trauma exposure. The DSO and BPD latent variables also were associated with exposure to adult non-interpersonal trauma, and the BPD variable was associated with exposure to adult interpersonal trauma. Thus, these findings suggest an incrementally increasing complexity of trauma exposure from PTSD to DSO, and from DSO to BPD. They also indicate that the psychopathology and psychosocial impairment involved in cPTSD/DSO is characterized by relational detachment and a perception of self as damaged, while BPD is characterized instead by a fragmented and unstable sense of self and impulsive relational dysregulation related to profound emotional emptiness and terror of abandonment.

In a sample of adults with histories of institutional child abuse, network analyses showed that PTSD and DSO symptoms were moderately interconnected [95]. In addition, dissociative and DSO emotion dysregulation symptoms were most central and most strongly connected to PTSD symptoms. On the other hand, BPD symptoms were only weakly inter-connected to one another, primarily centering on self-harm, chronic emotional emptiness, mood instability, and impulsivity. Furthermore, none of the BPD symptoms were more than weakly connected with either the PTSD or DSO symptoms, except to the PTSD symptom of reckless behavior. Thus, in this sample of adults who had extreme traumatic experiences of abuse, neglect, and separation from primary caregivers, dissociation and cPTSD’s chronic emotion dysregulation appeared to be the core symptoms with strong connections to other DSO and PTSD symptoms. In contrast, BPD symptoms occurred relatively independently of dissociation and both DSO and PTSD symptoms, except for PTSD’s reckless behavior (which is consistent with the BPD constellation of self-harm [61] and symptoms of extreme emotional and behavioral instability and alienation).

#### Summary

The person-centered research studies to date suggest that cPTSD’s DSO symptoms are distinct as a construct from the symptoms of both PTSD (see also [12]) and BPD, but also that the BPD symptoms tend to occur primarily in combination with PTSD and/or cPTSD/DSO symptoms rather than in isolation. Moreover, the combination of BPD, PTSD, and DSO symptoms is more strongly associated severe cumulative childhood interpersonal victimization than are either cPTSD/DSO or PTSD symptoms in the absence of BPD symptoms. Thus, in actual clinical practice BPD may occur without comorbid PTSD and cPTSD/DSO symptoms, just as the DSO features of cPTSD may occur without comorbid PTSD symptoms [12]. However, both the PTSD and cPTSD/DSO symptoms often occur with no BPD symptoms (except possibly BPD’s unstable sense of self and emotional emptiness), most often as a sequela of childhood emotional neglect (with or without childhood abuse).

However, despite the extensive co-occurrence of PTSD and cPTSD/DSO symptoms with those of BPD, the three syndromes thus appear to have distinguishing features. Hyperarousal and avoidance in reaction to intrusive re-experiencing of trauma memories are central to PTSD and have been found to be related to a persistent fear in the aftermath of the threat or actuality of death or unexpected loss in adulthood or childhood [3]. In cPTSD/DSO, intense diffuse emotional distress and self-perceptions of worthlessness, shame, and guilt are related to a sense of betrayal, fear of closeness, and severe emotional detachment in relationships. BPD, by contrast, is characterized by impulsive, intrusive, and angry enmeshment in, rather than only detachment from, primary relationships in reaction to a combination of a terror of abandonment and a fragmented and unstable sense of self, which is acted out as hostile and impulsive demands in relationships [30, 108, 127, 144]. Additional research is needed across a range of different clinical and general population samples to determine whether BPD or specific BPD symptoms can be consistently distinguished from cPTSD and its symptoms.

### The role of trauma and victimization in BPD and cPTSD

Adults diagnosed with BPD have been shown to be three times more likely than adults with other psychiatric or personality disorders to have experienced childhood maltreatment, and 13 times more likely than adults with no psychopathology [136]. Emotional abuse and neglect were particularly prevalent, with BPD associated with more than 30 times increased likelihood of the former and more than 20 times increased risk of the latter, as compared to non-clinical adult samples [136]. Physical abuse and neglect, and sexual abuse also were more prevalent for BPD cohorts than non-clinical controls, with an approximately seven times increased likelihood among BPD-diagnosed persons [136]. Childhood adversity was more than twice as likely to be reported by persons diagnosed with BPD than those with mood, psychotic, or any other personality disorders [136]. Extreme interpersonal trauma in adulthood (e.g., civilian exposure to war trauma, torture, sex trafficking, or genocide) also has been shown to be associated with severe personality disorders including BPD [120].

As noted above, emotional and physical neglect have been shown to be a unique risk factor for BPD [48, 136, 194]. However, emotional neglect also was found to be associated with cPTSD alone, and both emotional and physical neglect were associated with comorbid BPD/cPTSD in a sample of adults with sexual abuse histories [52]. Emotional abuse and neglect often occur in combination with physical and sexual abuse but have been found to have adverse biopsychosocial effects that are distinct from those of physical or sexual abuse [159, 165]. A sub-group of adolescents who had experienced emotional abuse alone was identified as distinct from other youths who had experienced sexual abuse and interpersonal rejection, physical abuse, and poly-victimization [26]. A meta-analysis of 124 studies found that childhood emotional abuse also was as strongly associated with suicide attempts, drug use, and sexually transmitted disease as neglect or physical abuse, and more strongly for depressive disorders [129]. A more recent meta-analysis with an international sample also indicated that emotional abuse may intensify the adverse effects of neglect and physical abuse on children’s behavioral problems [24].

Adults with BPD [14, 96, 98, 118, 121] and adolescents with BPD features [89, 154, 182] also are at increased risk for abuse or re-victimization as adults and for cumulative polyvictimization across the lifespan. Further, cumulative exposure to interpersonal violence over the lifespan was found to be associated with comorbid BPD/PTSD rather than either BPD or PTSD alone [83, 84]. Adult re-victimization following childhood maltreatment thus may be associated with the combination of PTSD and BPD – and whether this is particularly true when DSO symptoms also are present warrants investigation.

More specifically, sexual victimization in adulthood was found to be the most prevalent type of victimization among women with comorbid BPD/PTSD [156]. Consistent with that finding, childhood sexual abuse was shown to be the one type of adverse childhood experience that was strongly directly associated with both BPD and PTSD among adults in inpatient psychiatric treatment —[11]. Childhood sexual abuse has consistently been found to be a risk factor for BPD, and both childhood and adult sexual abuse and assault are associated with the most severe, lethal (e.g., self-harm), debilitating (e.g., dissociation), and chronic BPD symptoms [173] as well as with comorbid PTSD [27]. However, whether sexual abuse alone is associated with BPD and PTSD or cPTSD is not known, because sexual abuse often co-occurs with other forms of victimization that are associated with attachment insecurity and disorganization, including neglect, emotional abuse, and domestic violence [36, 37, 60, 171, 172].

On the other hand, childhood sexual abuse has been found to characterize a distinct sub-group of victimized young women in community populations who differ from other sub-groups whose members experienced either or both physical or emotional abuse but not sexual abuse [195]. A study with trauma-exposed children also identified a sub-group representing 15% of the sample who had experienced sexual abuse or assault between ages 6 and 12 years old but rarely any other type of victimization--whereas sexual abuse tended to occur in combination with other forms of abuse and victimization both earlier in childhood and in adolescence [57]. These findings raise the question of whether childhood sexual trauma is associated with adult BPD when it occurs alone, or only when BPD occurs in the context of other forms of victimization – and if the former is true, whether middle childhood/pre-adolescence may constitute a time of particular vulnerability to BPD due to a heightened risk of the occurrence of sexual abuse. Given the evidence cited above of common co-occurrence of PTSD/cPTSD symptoms when BPD symptoms are prominent, the question of whether those findings reflect a relationship between childhood sexual abuse with comorbid BPD/cPTSD rather than (or in addition to) with BPD alone warrants investigation.

Although a combination of emotional abuse with sexual or physical abuse has been found to be particularly strongly associated with severe childhood psychosocial problems in clinical samples, emotionally abused children and adolescents also have a profile of behavioral and emotional problems distinct from the symptom profile associated with sexual or physical abuse [159]. Among adults with sexual abuse histories, although childhood physical neglect and sexual or emotional abuse each were associated with comorbid BPD/ cPTSD, only emotional abuse distinguished the comorbid BPD/cPTSD sub-group from the cPTSD-only sub-group [52]. Those findings raise the question of whether childhood sexual abuse may be a risk factor for both cPTSD and BPD while emotional abuse may be a risk factor (alone, or more likely in combination with other forms of childhood maltreatment) for the particularly severe and impairing comorbidity of BPD/cPTSD.

#### Summary

Chronic and cumulative exposure to multiple types of interpersonal trauma, especially in formative developmental periods in childhood but also including in adulthood [35], has repeatedly been shown to have an incremental dose-response relationship to cPTSD [6, 12, 18, 20, 51, 52, 54, 75, 77, 87, 122, 155]. Childhood poly-victimization (i.e., four or more types of maltreatment) also has been found to be associated with both cPTSD alone and comorbid cPTSD/BPD [52]. Childhood emotional abuse and neglect [52] and sexual abuse [156] also may be of particular importance in the development of cPTSD or PTSD when they are comorbid with BPD—and this comorbidity of trauma-related disorders with BPD may occur more often than BPD in the absence of PTSD or cPTSD [52, 73, 83, 84].

### Can BPD be distinguished on a neuropsychological basis from PTSD and cPTSD?

With the many unanswered questions about the role and type of trauma exposure in the etiology of BPD, cPTSD, and PTSD, psychobiological studies exploring commonalities and distinguishing features of the disorders are particularly important. Although several recent relevant studies have been reported, it is important to note that cPTSD as defined by the *ICD-*11 has not been included in any published neuroimaging studies to our knowledge. Distinctions in neural patterns that have reported between BPD and cPTSD thus are based mainly on a DESNOS formulation of cPTSD, or using severe child-abuse-related *DSM-*5 PTSD as a proxy for cPTSD [71]. Neuroimaging and psychobiological research comparing the *ICD-*11 version of cPTSD to BPD clearly is needed.

A case-control study with 45 adult outpatients diagnosed with BPD and 56 matched non-psychiatric controls [170] found that BPD was associated with deficits in verbal comprehension, sustained visual attention, working memory and processing speed. Comorbid BPD/PTSD was associated with additional deficits in high-order neurocognitive functions (i.e., verbal comprehension, visual episodic memory, perceptual reasoning). Although the BPD patients reported more severe childhood maltreatment than controls (e.g., 75, 62 and 49%, respectively, reported moderate to severe emotional abuse, emotional neglect, and sexual abuse or physical neglect), childhood trauma was associated only with one neurocognitive deficit—and only with trauma in the form of physical abuse.

Another case-control study with 21 BPD patients and 14 healthy controls [137] found evidence among the BPD cohort of lower functional connectivity within the brain’s Salience Network (i.e., the limbic stress reactivity and PfC executive function sites) and the Default Mode Network (DMN) (i.e., midline sites from front to back of the brain that are involved in self-referential processing). Reduced connectivity in the salience network was associated with impairment in higher order cognitive abilities and in the ability to inhibit interpersonal aggression. Reduced connectivity in the DMN was associated with heightened levels of anger and its interpersonal expression.

A case-control study with 26 adolescents (23 girls) diagnosed with BPD and 25 non-clinical controls found that the severity of emotional and physical abuse, neglect, and sexual abuse were associated with the severity of BPD symptoms, and higher order reflective function deficits (i.e., an aspect of executive control) partially mediated the relationship between emotional abuse and BPD symptom severity [138]. A meta-analysis similarly concluded that a combination of childhood emotional abuse and neglect with limited executive control in adulthood was associated with the development of BPD, and that previously maltreated adults with higher levels of executive control tend to experience relational mistrust and self-blame (which are characteristic of cPTSD/DSO) rather than BPD [48].

Individuals with BPD have been shown to exhibit dorsolateral PfC hypo-activation and orbital PfC and amygdala hyperactivation and connectivity in response laboratory tasks that elicit negative emotions [153]. In BPD, deficits in interpersonal trust, tolerance of aloneness, and recognition of conventional norms of social cooperation/fairness also have been documented and shown to be associated with altered patterns of anterior cingulate, temporal lobe, and insula activation [97, 149]. These specific forms of interpersonal dysregulation may be distinct to BPD, but whether they are moderated by either childhood maltreatment or PTSD remains to be determined [97].

However, unlike the biologically based emotion dysregulation that characterizes fear-related syndromes such as PTSD, BPD may involve altered brain connectivity associated with intolerance of emotional distress. This is consistent with findings that BPD occurs in adults who have experienced childhood maltreatment primarily but only if they have limitations in the kinds of executive control necessary to modulate emotional distress [48].

Correspondingly, individuals with a BPD diagnosis or symptoms have been shown to have structural brain abnormalities (i.e., increased gray matter volume) in prefrontal cortex areas involved in cognitive control [128] and alterations in endocannabinoids potentially associated with reduced distress tolerance [147].

Whether similar structural brain abnormalities characterize cPTSD is not yet known. The research showing that higher levels of executive control are associated with mistrust and self-blame rather than with BPD [48] raises an intriguing possibility. Those clinical features (i.e., mistrust, self-blame) are core features of the interpersonal dysregulation and negative self-perceptions features of DSO. Thus, it could be that cPTSD is a sequelae of childhood maltreatment (or other extreme forms of interpersonal victimization at any point in the lifespan, Brewin et al., 12,017) for individuals who have relatively intact executive control capacities and the neurobiological connectivity and capacities that are its infrastructure.

Relatedly, a meta-analysis of neuroimaging studies testing reactions to affective stimuli that were negatively-valenced versus ones that were neutral found that BPD was associated with hyperactivation of limbic (i.e., amygdala and hippocampus), posterior cingulate, temporal cortical, and medial PfC areas and hypoactivation of the postcentral gyrus [153]. Major depression was associated with hyperactivation of the insula and thalamus, and hypoactivation of the amygdala, and the temporal and parietal gyri. PTSD was associated with hyperactivation of the orbital PfC, amygdala, striatum, and temporal gyrus, and hypoactivation of the postcentral gyrus. PTSD also showed greater levels of activation than BPD in the dorsolateral PfC, amygdala, temporal gyrus, posterior parietal gyrus, and premotor cortex, and than major depression in the amygdala, hippocampus, and ventrolateral and dorsolateral PfC. BPD had higher levels of activation than major depression in the amygdala, hippocampus, and ventrolateral PfC. Thus, BPD and PTSD were distinguished from healthy controls and major depression by activation of the amygdala and hippocampus in reaction to stimuli eliciting negative emotion, although differing in that in BPD the brain activity appeared to reflect conscious emotion processing (i.e., left amygdala) while automatic emotion processing was implicated in PTSD (i.e., right amygdala) [153].

Both BPD and PTSD also shared a tendency to activate the ventrolateral PfC—an area associated with attempts to suppress excess emotion reactivity—in response to negatively-valenced affective stimuli [153]. However, deficits in both inhibition and regulation of hyperactivation by the amygdala and of heightened states of negative emotion have been linked specifically to BPD in the context of a history of childhood adversity [8]. Thus, BPD may involve the failure of conscious attempts to inhibit emotional reactivity that occurs in relation to perceived or actual interpersonal rejection or abandonment, whereas PTSD may involve the failure of habitual non-conscious attempts to inhibit fear due to perceived or actual threats of physical or relational harm.

PTSD was found to differ from BPD (and major depression) in enhanced activation of the dorsolateral PfC (which is implicated in cognitive coping) and the striatum (which is involved in identifying and preparing for threats, especially following unanticipated aversive outcomes) [153]. Additionally, PTSD differed from BPD (and major depression) in showing reduced activation of the ventral premotor and right posterior parietal cortices, which inhibit impulsivity and support attentional and cognitive processes regulating negative emotions [153]. Potentially further contributing to difficulties in regulation of negative emotions, BPD also was associated with lesser inhibitory PfC activation in reaction to negative affective stimuli than PTSD. All three disorders showed increased activation in the DMN, a system that tends to be disconnected from the PfC in PTSD [2] and to be hypoactivated in executive function tasks in BPD [1]. Thus, self-focused distress taking the form of rumination or intrusive re-experiencing may be pronounced in both BPD and PTSD. However, unlike the relatively unfettered nature of rumination in BPD, in PTSD there may be at least partially successful attempts to regain cognitive control through the activation of the PfC.

cPTSD was not assessed in the studies reviewed by Schulze et al. [153], raising the question of whether the deficits in amygdala inhibition and emotion regulation are associated with cPTSD/DSO rather than, or in addition to, BPD or PTSD per se*.* Although both DESNOS and BPD have been found to be associated with a hippocampally-mediated bias toward memory encoding of negative (vs. positive) information [169], in cPTSD there is evidence of heightened dorsolateral and ventromedial PfC and anterior cingulate activation in reaction to negatively valenced emotional stimuli [71, 168]. This heightened prefrontal activation is associated with enhanced executive control and emotion regulation similar to that found in PTSD but the opposite of the diminished PfC inhibition found with BPD [153]. Thus, cPTSD and PTSD involve the at least partially successful deployment of executive control in an attempt to inhibit emotional distress to an extent that is not possible for persons with BPD. Further, the consequences of functional deficits in prefrontal modulation of amygdala hyperactivity may be associated with different interpersonal/affective contexts in cPTSD (e.g., betrayal of trust) than in PTSD (e.g., physical or emotional threat) or BPD (e.g., rejection or abandonment).

Another potential neurobiological difference between BPD and PTSD involves pain. Although both BPD and PTSD have been found to be associated with complaints of heightened physical pain, they appear to have distinct pain profiles [150]. BPD often involves pain analgesia related to the use of dissociation, both in current and (when exposed to stressors) remitted BPD cases [17]. However, among patients in treatment for chronic pain, BPD was associated with a history of childhood maltreatment and both acute hypersensitivity to emotion-related pain and chronic central nervous system hypersensitivity [82], consistent with increased attempts to self-medicate through the use of analgesic medications [49]. In PTSD, pain-related impairment due to hyperalgesia rather than analgesia also is prominent [7, 38, 141]. This is consistent with neuroimaging findings of an pattern of sustained amygdala/limbic activation with reduced connectivity to PfC inhibitory areas [102] and strong bilateral connections between the locus of affective pain recognition in the cortex (the anterior insula) and the midbrain locus of defensive states and endogenous opioid release (the periaqueductal gray) [65, 166] in PTSD.

However, a dissociative sub-type of PTSD (PTSD/D) has been identified [101] that is associated with analgesia rather than hyperalgesia. Analgesia in PTSD/D potentially is related to deficits in self-awareness resulting from strong connectivity between the posterior and anterior insula with posterior cortices (e.g., precuneus, lingual gyrus) associated with implicit (nonconscious) visuospatial memory [65]. The neurobiology of pain in PTSD is not well understood, although PTSD and chronic physical pain appear to be mutually exacerbating in a bidirectional manner [93].

Whether cPTSD has a profile analgesia or hyperalgesia, with corresponding brain structures and activation or connectivity patterns, has not been investigated.

#### Summary

While preliminary, taken together these findings suggest that emotion and relational dysregulation in BPD may involve brain alterations associated with unstable and ruminative self-awareness, fear of abandonment, inability to recover from intense negative affect, and, in some cases, alternating hyperalgesia and dissociative analgesia. In contrast, PTSD secondary to childhood maltreatment, which actually may be cPTSD/DSO rather than PTSD alone in many cases, appears to involve brain alterations related to stable dysphoric self-perceptions, generalized hypervigilance, avoidance of (and possibly habituation to) chronic pain and negative emotion states, detachment from relationships, and, in a sub-set of cases, dissociative fragmentation of consciousness and alternating states of hyperalgesia and analgesia. The neurobiological differences between BPD and childhood maltreatment-related PTSD/cPTSD thus parallel the phenomenological and diagnostic differences between the disorders.

This confluence of evidence raises a question; given their similar traumatic antecedents and frequent co-occurrence in person-centered research studies, why do BPD and cPTSD not have more similar neurobiological substrates and clinical phenomenologies? Two domains prominent in both BPD and cPTSD will be considered next in order to address that question: (1) disorganized attachment and emotion dysregulation and (2) pathological dissociation.

### Disorganized attachment and emotion Dysregulation in cPTSD and BPD

The combination of childhood abuse and neglect by primary caregivers, which is an antecedent for both cPTSD and BPD, constitutes a significant disruption to the development of the secure emotional bonds that are the basis for internal working models of attachment security [13]. The threat to physical (and sexual) safety caused by abuse and neglect also constitutes a potentially traumatic stressor [25]. Attachment insecurity in infancy has been shown to be predictive of emotion regulation decades later in adulthood [55]. When primary caregivers are either anxious/intrusive on one hand or detached/non-responsive on the other, while otherwise being fairly consistent in their childrearing, the child’s internal attachment working models (i.e., core affective/cognitive schemas about intimacy, nurturance, and protection) tend to be insecure (i.e., anxious/pre-occupied or detached/avoidant).

A more serious condition occurs when caregivers are both a desired but unavailable or unpredictable source of comfort/security and a source of danger or abuse to the child, often in confusing ways. The child’s attachment working models in this case of this type have been described as disorganized. These children simultaneously seek closeness and protection (approach) to caregivers as a biological survival mechanism while withdrawing (avoidance) due to feeling fearful of being hurt or abandoned by caregivers who are a source of danger or who may be unavailable or unwilling to provide nurturance and protection [112]. Disorganized attachment in infancy and middle childhood has been found to subsequently predict BPD symptoms in adolescence and young adulthood [110]. Adolescents diagnosed with BPD also have been found to have internal working models characterized by disorganized attachment, in contrast to non-clinical youth who had predominantly secure working models [116].

When a child’s sense of attachment becomes chronically disorganized, lifelong problems with relationships, the core sense of self or identity, suicidality, and addictions may develop [80, 104, 114, 164, 167]. These problems are related to deficits in core psychological capacities for emotion regulation that emerge in infancy and that are consolidated in childhood when secure attachment bonding occurs [16, 145]. In adolescence, disorganized attachment has been shown to be associated with heightened functional connectivity between the amygdala and areas in the brain involved in visual and self-referential (i.e., DMN) processing, but reduced connectivity with the inhibitory PfC [181]—similar to findings with adults diagnosed with BPD. Disorganized attachment also was found to be associated with reduced white matter integrity among adolescents in areas of the brain that connect the two hemispheres (i.e., corpus callosum) and the PfC longitudinally to the temporal, parietal, and occipital cortices (e.g., the DMN) [140]–also similar to findings for BPD and several other psychiatric disorders including PTSD.

When disorganized attachment persists into adulthood, emotion dysregulation consistent with cPTSD (e.g., withdrawal, rumination, inability to self-calm) and with BPD (e.g., desire for retribution, instability, hostility) has been shown to be more severe than for adults with secure, anxious, or avoidant attachment working models [53]. Consistent with those findings, neurobiological research shows that adults with disorganized attachment working models tend to experience deactivation in areas of the temporal and parietal cortices in the brain that are involved in social cognition and empathy, in contrast to heightened activation of those areas in the brain by other adults [133]. Disorganized attachment in infancy also has been shown to predict larger amygdala volume (and potentially heightened emotional reactivity and dysregulation) in adulthood [111].

With respect to BPD specifically, young adults diagnosed with BPD were found to **be** eight times more likely than other young adults (with or without other psychiatric diagnoses) to engage in disorganized attachment interactions with a parent [92]. Young adults from a non-clinical but lifetime adversity sample who engaged in interactions with a parent in a manner consistent with disorganized attachment were found to be likely than peers to have BPD symptoms and to engage in non-suicidal self-harm, although this was unrelated to their child abuse histories [109]. Adults in mental health treatment were found to display two different types of disorganized attachment: a highly emotionally labile group whose members had the most severe BPD symptoms, and an emotionally detached group whose members had the most severe avoidant and schizoid symptoms (although trauma history and PTSD symptoms were not assessed in that study) [5]. Among adults in mental health treatment, insecure or disorganized attachment and emotion dysregulation have been shown to be inter-related and to mediate the relationship between childhood maltreatment and BPD features [130]. Thus, disorganized attachment may be associated with BPD or cPTSD or both, and more specifically, with the severe emotion dysregulation found in these disorders.

Emotion dysregulation in BPD takes the form of “affective instability … intense episodic dysphoria, irritability, or anxiety” and “chronic feelings of emptiness” in BPD ([3], p. 663). In adults with BPD, emotion dysregulation, particularly involving anger [56] in a context of perceived rejection [4, 48, 132], also has been shown to be associated with both prior victimization by child maltreatment and subsequent violence perpetration toward self and others (primarily intimate partners) [73, 79, 96, 98, 107, 126]. Emotion dysregulation also was shown to mediate the relationship between a history of childhood emotional abuse and current fear of rejection with adults’ BPD symptoms [47]. Among adolescents in psychiatric residential treatment, emotion dysregulation has been shown to mediate the relationship between childhood interpersonal violence exposure and BPD (and also PTSD) symptoms [14]. BPD also involves emotion dysregulation associated with deficits in social cognition related to dichotomous thinking, paranoia, aggressive attributions, and increased attention to, but impaired recognition, understanding, and empathy for, the emotions, thoughts, and intentions of others [30, 108, 127, 144].

Although the research base is more limited regarding emotion dysregulation and cPTSD, it has been shown to differ from the emotional dysregulation found in BPD. In cPTSD, emotion dysregulation is characterized by trauma-related self-perceptions (e.g., guilt, shame, helplessness), fear of closeness in relationships, and numbing or suppression of emotional expression [88]. Emotion dysregulation has been found to adversely contribute to the psychosocial impairment experienced by adolescent victims of sexual assault over and above the effects of PTSD and other cPTSD/DSO symptoms [184]. Emotion dysregulation also has been found to mediate the relationship between a history of childhood maltreatment and other cPTSD/DSO symptoms in adults in mental health treatment [94]. Although the clinical phenomenology of emotion dysregulation differs for cPTSD and BPD, emotion dysregulation appears likely to be a link between childhood maltreatment adult symptoms and impairment in both cPTSD and BPD.

The similarities and distinctions between emotion dysregulation in BPD and cPTSD, and whether emotion dysregulation mediates the relationship between childhood victimization and adult BPD and cPTSD, have been investigated in a series of studies with a cohort of adult psychiatric inpatients who met criteria for BPD with or without a comorbid somatoform disorder, or with a somatoform or other severe Axis I disorder without BPD [175, 177, 179]. BPD was found to be primarily associated with under-regulation of emotion associated with a fear of abandonment, while cPTSD (as defined by DESNOS) involved alternating states of under-regulation of emotion combined with trauma-related positive dissociative symptoms (i.e., flashbacks, identity fragmentation) or over-regulated emotion combined with negative dissociative symptoms (i.e., derealization, depersonalization, fugue states, physical conversion reactions [29, 176, 178, 180].

#### Summary

BPD involves a generalized under-regulation of intense distress related to real or perceived abandonment or rejection, whereas emotion dysregulation in PTSD is characterized by attempts to over-regulate (e.g., emotional numbing, avoidance, dissociation) distress related to reminders of traumatic experiences. In cPTSD (as defined by DESNOS), emotion dysregulation may take the form of either under- and over-regulation of trauma-related distress, primarily related to trauma-impacted beliefs about self and relationships (e.g., guilt, shame, helplessness, fear of closeness). Research is needed to determine how attachment disorganization and subsequent emotion dysregulation are associated with cPTSD (as assessed with the *ICD-*11 criteria) and BPD.

### Dissociation in BPD, PTSD, and cPTSD

Attachment disorganization [15, 115, 119] and emotion dysregulation [68, 69, 119, 188] both have been shown to be related to pathological dissociation. Dissociation, in turn, is recognized as a symptom of BPD (as a transient reaction to extreme stress) and the PTSD dissociative sub-type (PTSD/D) [3]. Dissociation was a symptom in initial formulations of cPTSD, but was not included as a cPTSD symptom in the *ICD-*11 because of research indicated that dissociative symptoms did not load on factors derived empirically from assessments based on those earlier versions of cPTSD [151]. Dissociation also has been shown to have a brain functional connectivity signature involving frontoparietal and DMN activation that is distinct from that of both child maltreatment and PTSD [103]. Dissociation thus was not included in subsequent structural analysis studies of the symptoms selected for the *ICD-*11 version of cPTSD ([12], p. 9).

However, dissociation warrants consideration when comparing BPD and cPTSD because the trauma-related antecedents of those disorders (i.e., childhood maltreatment and disorganized attachment), and their central clinical feature (i.e., emotion dysregulation) are complexly intertwined with dissociation. For example, a study with pre-school age children, including both victims of sexual abuse and a non-abused comparison group, found that sexually abused children’s self-reported attachment disorganization and parent-observed emotion dysregulation were strongly inter-related, more severe than for the non-abused children, and mediated the relationship between sexual abuse and parent-observed dissociation 1 year later [67]. Among young adults, disorganized attachment interactions with a parent were shown to mediate the relationship between child abuse history and pathological dissociation [15]. Whether dissociation in the aftermath of childhood maltreatment, disorganized attachment, and the development of persistent emotion dysregulation plays a role in BPD, PTSD, and cPTSD or can contribute to their differentiation, therefore warrants consideration.

Dissociation has been shown to be problematic for a sub-set of the individuals who are diagnosed with BPD, PTSD, and cPTSD, both as freestanding disorders and when comorbid with one another [95, 179, 180]. “Transient dissociative symptoms related to extreme stress and real or imagined abandonment” ([3], p. 664) are cardinal symptoms of BPD, although tending to occur primarily for BPD-diagnosed individuals who have a history of childhood abuse [91, 119]. A meta-analysis found that patients diagnosed with BPD reported more severe and extensive symptoms of dissociation than those diagnosed with other psychiatric disorders except for PTSD [146]. Both a history of childhood interpersonal trauma and current dissociative symptoms—specifically neurological conversion symptoms—were found to be associated with cortical activation patterns characteristic of emotion dysregulation (i.e., frontal EEG asymmetry) among adults in treatment for BPD when they were exposed to aversive pictures [134]. In another study, adults diagnosed with BPD were induced to experience dissociation and then engaged in activities involving executive control; the BPD patients showed reduced emotional working memory and reduced activation of areas in the brain associated with emotional (e.g., amygdala, cuneus, lingual gyrus) and self-referential (e.g., posterior cingulate in the DMN) processing, and altered functional connectivity between those and other areas involved in emotion awareness, memory, and regulation and self-awareness were observed [96, 98]. Thus, dissociation in BPD may occur as a non-conscious compensation for failed attempts at self-awareness and executive control in states of severe emotion dysregulation.

Although dissociative symptoms are highly correlated with PTSD symptom severity among adults decades after experiencing childhood sexual abuse [123], pathological dissociation was not included in the PTSD diagnosis until the *DSM-*5 revision identified a dissociative sub-type of PTSD (PTSD/D) [100, 101]. A study with military veterans and active duty personnel diagnosed with PTSD found that women (but not men) who met criteria for PTSD/D were more likely to meet criteria for BPD (and also for avoidant personality disorder) than those with traditional PTSD [189]. Among adolescents involved in juvenile justice, dissociation and PTSD symptoms were found to separately mediate the relationship between poly-victimization and internalizing problems [42]. Based on these findings, PTSD and BPD appear distinct with regard to dissociation (i.e., transient reactions to extreme interpersonal distress in BPD versus chronic adaptation to fear related to poly-victimization in PTSD), *except* for possible overlap in some cases in which PTSD/D and BPD co-occur [189].

Similar to PTSD, cPTSD has been found to be associated with heightened levels of dissociative symptoms among adults in mental health treatment [32, 33]. Moreover, patients meeting criteria for cPTSD have been shown to have more severe dissociative symptoms than patients meeting criteria for PTSD without DSO [74]. In a study with adult mental health patients, psychoform (but not somatoform) dissociation partially mediated the relationship between a history of childhood maltreatment and cPTSD (assessed using DESNOS criteria) [176]; importantly, this mediation occurred independent of the effects of BPD symptoms, fears of abandonment and closeness, and emotion dysregulation [180]. Further analyses with data from that same psychiatric clinical sample demonstrated that psychoform dissociation also mediated the relationship between a history of childhood maltreatment and BPD, independent of the effects of cPTSD symptoms, affect dysregulation, and fears of abandonment and closeness [179]. In the latter study, a direct relationship was found between childhood sexual abuse and BPD, independent of the effects of dissociation, emotion dysregulation, relational fears, and cPTSD [179].

#### Summary

These findings suggest that pathological dissociation may occur in PTSD (i.e., the dissociative sub-type), cPTSD (as an associated symptom), or BPD (in extreme states of emotional dysregulation). Thus, dissociation appears to play different roles in the three disorders, although research is needed to determine the exact nature of its involvement in each disorder. Dissociation also appears to be involved in the relationship between childhood maltreatment with both cPTSD and BPDin a manner that is distinct from either disorganized attachment or emotion dysregulation. Here again, replication with cPTSD assessed using the *ICD-*11 criteria is needed in order to better understand the similarities and differences in the role played by dissociation in BPD and cPTSD, and to clarify the complex interrelationships of attachment disorganization, emotion dysregulation, and dissociation in those disorders.

### Implications for clinical practice and research

Research over the past decade has provided increased clarity about the distinction between cPTSD and BPD, although many questions remain to be answered. In terms of clinical assessment and treatment, the phenomenological and neurobiological signatures of BPD and cPTSD suggest that different forms of emotion dysregulation may evolve in the aftermath of childhood maltreatment and disorganized attachment. In the research arena, there is a need for more systematic investigation of the inter-relationships of the component processes involved in adaptation to childhood maltreatment, attachment disorganization, emotion dysregulation, and dissociation that are associated with cPTSD and BPD. More specific implications for clinical practice and research now will be discussed.

### Assessment and diagnosis

As part of a trauma-informed intake and assessment/diagnosis, clinicians evaluating clients who present with prominent symptoms of BPD should not overlook the possibility that not only PTSD but also cPTSD/DSO symptoms amay be present as an additional complexity or exacerbator of symptomatic and impairment severity. Moreover, cPTSD also may better account for some symptoms that have been attributed to BPD (e.g., if negative self-perceptions are stable rather than unstable, or if a sense of emptiness is due to emotion numbing or detachment from relationships rather than to fundamental existential alienation). Considering cPTSD is especially relevant if there is a history of childhood maltreatment, poly-victimization, disorganized attachment, or chronic dissociation [42, 46, 183].

The potential role of cPTSD also should be considered when emotion dysregulation attributed to BPD involves distress related to a generalized sense of physical or emotional threat or betrayal, instead of (or in addition to) a terror of real or perceived abandonment or interpersonal rejection. This is especially the case when there is evidence of withdrawal or detachment in primary relationships instead of (or in addition to) enmeshment, hostility, and intrusive demands on significant others. In order to identify these potential markers for cPTSD, it is necessary to include a systematic inquiry about the patient’s history of engagement in relationships as well as of maltreatment, of other forms of victimization and traumatic stressors (including traumatic losses), as well as of developmental experiences that indicate attachment insecurity and disorganization beginning in early childhood, and of aspects of emotion dysregulation and both psychoform and somataform dissocation.

Such highly sensitive personal historical inquiry can be emotionally destabilizing for individuals with BPD, who are by definition prone to emotional lability, identity instability, perceiving significant others as rejecting and abandoning, and transient severe dissociation. However, when done in calm, empathic, accepting, and nonjudgmental manner, assessment of personal history (including maltreatment and other potentially traumatic experiences) can not only identify trauma-related precursors of current symptom but can also enable the patient and therapist to construct a chronological narrative that reveals how some BPD (and cPTSD) symptoms emerged as logical adaptations to traumatic exposure and adversity [40]. Such a collaborative person-centered and trauma-focused assessment [22, 23] also can enhance the often tenuous therapeutic alliance with patients presenting with BPD [59, 81], by signifying genuine interest in the patient’s lived experience and willingness to assist the patient in maintaining or regaining emotional regulation when engaging in therapeutic work.

Assessment instruments for cPTSD have become increasingly available over the course of the past several decades and can be used selectively by the clinician [158]. Empirically validated cPTSD interview guides have been developed, including the International Trauma Questionnaire (ITQ) for adults [21] and children [66], and the Developmental Trauma Disorder Semi-structured Interview (DTD-SI) for children and adolescents [46, 174]. With the comprehensiveness and precision afforded by these assessment guides, it has become increasingly possible for clinicians (and researchers) to identify the symptoms of cPTSD and distinguish them for purposes of differential diagnosis from symptoms of BPD [34].

### Psychotherapy

A systematic review of 75 randomized clinical trials testing psychotherapy for BPD [163] identified 16 approaches to treatment, with Dialectical Behavior Therapy [105, 106] and Mentalization-Based Therapy (MBT) [185] most often the focus. Overall, despite evidence of larger reductions in BPD symptom severity by active psychotherapies than treatment as usual, the average reduction of 3.6 points on a 0–36 scale was only slightly higher than the threshold for a minimal clinically relevant difference (MIREDIF; 3.0 points). Moreover, despite evidence of reduced suicidality, self-harm, and depression, and improved psychosocial functioning, the observed benefits were less than those required by MIREDIF. A detailed analysis of outcomes of a randomized clinical effectiveness study showed that DBT resulted in greater reductions in experiential avoidance and expressed anger than community-based expert treatment, but not for guilt, shame, anxiety or anger suppression and dyscontrol [125]. Controlled evaluations of MBT also have not shown evidence of its superiority compared to treatment as usual or supportive psychotherapy in the domains of anxiety, depression, social adjustment, and relational functioning [185]. These symptoms are quite similar to the symptoms of PTSD and cPTSD, which suggests that even the BPD psychotherapies with the strongest empirical support may be enhanced by the addition of therapeutic modalities that directly target the symptoms of PTSD and cPTSD /DSO.

Fortunately, advances in empirically supported treatment for comorbid PTSD and BPD have been reported recently. Dialectical Behavior Therapy (DBT) was adapted with a modified form of Prolonged Exposure (PE) therapy for PTSD (DBT + DBT PE) and pilot tested with women diagnosed with comorbid BPD and PTSD [63]. Treatment was provided for 1 year (*M* = 29 vs. 39 individual therapy sessions and 24 vs. 29 skills group sessions for DBT and DBT + DBT PE, respectively). Compared to DBT alone, treatment completers in DBT + DBT PE had greater and more stable improvement in PTSD symptoms and 80% remission from PTSD (versus 40%), and a lower likelihood of suicide attempts or self-harm, as well as larger reductions in dissociation, trauma-related guilt, anxiety, shame, depression. DBT + DBT PE also was associated with improvement in social adjustment, health-related quality of life, and overall functioning—but only after PTSD symptoms and related cognitions were reduced [62, 64].

However, DBT + DBT PE was not superior to DBT in the interpersonal problems or overall quality of life domains [63]. Also, more than 40% of participants dropped out prematurely, and fewer than half (8 of 17) of those randomized to DBT + DBT PE actually received PE due to dropping out or not being stably non-suicidal and non-self-harming and being willing and able to experience intense emotions. PE was not initiated until on average 22 weeks of DBT had been completed: thus, DBT was utilized as what has been described in the cPTSD treatment field [23] as a Phase 1 safety/stabilization intervention prior to initiating Phase 2 trauma memory processing, and half of the participants did not progress beyond Phase 1 to receive PTSD-focused treatment.

A subsequent report with a larger sample of women receiving DBT + DBT PE (*N* = 30) added a third phase focused on addressing remaining BPD-related problems in psychosocial functioning [62, 64]. Almost half (40%) of the DBT + DBT PE recipient did not move beyond Phase 1, and 30% of those who began PE did not complete it. PTSD was found to improve only when trauma memory processing was initiated. Although DBT + DBT PE was associated with higher levels of general well-being than DBT alone, DBT + DBT PE was not associated with significantly greater reductions than DBT alone in PTSD, BPD, or dissociation symptoms or urges to commit suicide or self-harm. Thus, adding PTSD trauma memory processing to DBT had some benefits, but most women receiving that intervention either dropped out or had limited improvement in PTSD or BPD symptoms.

An alternative adaptation of DBT (DBT-PTSD) was developed and tested against an evidence-based PTSD psychotherapy (Cognitive Processing Therapy, CPT) tested in a large randomized clinical trial [9]. DBT-PTSD supplemented standard DBT with two variants of PTSD trauma memory processing (Cognitive Therapy and PE), Compassion Focused Therapy [193], and Acceptance and Commitment Therapy [139]. Women diagnosed with PTSD related to childhood abuse who met at least three (*M* = 5, which is the threshold for a *DSM-*5 BPD diagnosis) of the nine symptom criteria for BPD (including affective instability) were randomly assigned to DBT-PTSD (*N* = 98) or CPT (*N* = 98). They received comparable amounts (up to 45 sessions) of treatment over the course of a year, followed by three sessions over the next 3 months. Significantly fewer women dropped out prematurely from DBT-PTSD (25.5%) and CPT (39%). Both treatments resulted in reductions in PTSD symptoms, but those improvements and PTSD remission and reliable change were greater in DBT-PTSD and only DBT-PTSD resulted in reduced dissociation, depression, and BPD symptoms.

A third study of an intervention combining an evidence-based PTSD psychotherapy (Eye Movement Desensitization and Reprocessing, EMDR) with DBT is underway at the time of this review, in a randomized clinical trial with patients with PTSD and comorbid full and subthreshold BPD [157]. Thus, the three most extensively tested approaches to PTSD psychotherapy (EX, CT, and EMDR) have been systematically integrated with DBT. Whether similar blended or concurrent approaches to MBT are feasible and effective for comorbid BPD/PTSD is a logical next step along these lines.

With recent evidence from clinical practice [44], and from a meta-analysis [86], showing that psychotherapies originally designed to treat PTSD can be successfully adapted clinically to effectively treat cPTSD [85], clinicians and researchers also can draw on these cPTSD-adapted approaches to psychotherapy when treating the large sub-group of patients with BPD who have comorbid cPTSD/DSO and traditional PTSD symptoms. However, the meta-analysis found limited data on the benefits of evidence-based therapies for PTSD in reducing emotion dysregulation, and that childhood-onset trauma was associated with poorer therapeutic outcomes [86]. Karatzias and Cloitre et al. [20] recommend a flexible modular approach to psychotherapy for cPTSD in which evidence-based interventions are deployed in a sequence that is individualized to address each patient’s primary impairments.

In light of the impairment experienced due to dissociation by many patients diagnosed with BPD and cPTSD, the incorporation of therapeutic approaches to the treatment of dissociative disorders (DD) also warrants consideration [161]. No single manualized model of psychotherapy for DD has been empirically validated, but the Treatment of Patients with Dissociative Disorders (TOPDD) study has been conducted with a series of prospective longitudinal investigations that address the gaps in research about treating individuals with DD. A stabilization-focused, trauma-informed, staged approach to treatment was found to be associated with a wide range of improvement in DD patients’ symptoms, daily functioning, emotion regulation, and quality of life, as well as decreased hospitalizations and treatment costs [124]. An on-line education program for therapists and their DD-diagnosed patients has been adapted from the TOPDD study findings, providing a basis for both clinicians and clinical researchers to incorporate the treatment guide in clinical practice and clinical trials research with patients with BPD and severe dissociation.

### Research

The scientific challenges related to BPD and cPTSD continues to fall in the domains identified by Ford and Courtois [44]: to systematically identify the biopsychosocial *diatheses* (e.g., genetic/familial/ neurobiological risk/protective factors), (2) *stressors* and *adversities* (e.g., maltreatment, traumatic victimization, absent or poorly attuned caregiving) and *developmental trajectories* (e.g., disorganized attachment, chronic affect dysregulation, pathological dissociation) that are unique to or shared by BPD, PTSD, and cPTSD..

Understanding the childhood origins and pre-adult course of cPTSD and BPD is crucial to the development of early intervention and prevention. Based on research on the childhood sequelae of maltreatment and poly-victimization [25] on an international survey of clinicians [45], and results from a field trial study with both clinical and non-clinical samples of children [160, 174], developmental trauma disorder (DTD) was formulated as a pediatric variant of cPTSD. DTD involves three domains: four symptoms of psychological or somatic *emotion dysregulation*, five symptoms of threat-related *cognitive-behavioral dysregulation*, and six symptoms *of self-relational dysregulation*. DTD addresses the core domains of both cPTSD and BPD, potentially serving as a precursor to either disorder. However, DTD includes symptoms that differ from those of cPTSD and BPD as well.

The DTD emotion dysregulation symptoms include the adult cPTSD/DSO symptoms of intense distress and anger but also include symptoms found in BPD (i.e., difficulty recovering from intense distress) and symptoms distinct from both cPTSD/DSO and BPD (i.e., somatic expression of distress, and unawareness of or inability to express emotions or bodily states—the latter can involve dissociative states, but unlike the BPD dissociative symptom these may be chronic rather than transient and do not involve paranoia). The DTD cognitive–behavioral domain has symptoms found in BPD and *DSM-*5 PTSD (i.e., reckless or self-endangering behavior, self-harm), but also developmentally-attuned childhood symptoms that are not included in BPD or cPTSD (i.e., maladaptive self-soothing, deficits in goal directed behavior). The DTD self-relational domain includes negative self-perceptions that differ from those in cPTSD and BPD (i.e., stable view of self as damaged, versus unstable and guilt- or worthlessness-focused self-perceptions). In the relational domain, DTD has symptoms similar to cPTSD’s detachment and avoidance and BPD’s attachment disorganization, defensive aggression, relational enmeshment, and deficits in empathy and relational boundaries (the latter including boundary violations in an attempt to avoid abandonment, as well as excessively impermeable boundaries) —but also excessive empathy, which is found in neither BPD nor cPTSD.

Thus, DTD includes symptoms that closely parallel most of the symptoms of adult BPD and cPTSD/DSO as well as symptoms that are developmentally specific to childhood and related to children’s adaptations to traumatic victimization and disrupted attachment bonding [25] that could be precursors of adult cPTSD or BPD. However, DSO symptoms also have been empirically identified in children [66, 143], and adolescents and young adults [122, 131]. Adult BPD also may be preceded by BPD in adolescence [194], or by BPD-like symptoms that manifest earlier in childhood among adolescents and adults diagnosed with BPD (e.g., self-harm; disorganized controlling behavior; regulatory problems) [110, 187, 191].

## Conclusions

Although BPD and cPTSD/DSO overlap substantially in their defining symptoms, childhood maltreatment antecedents, and neurobiology, the syndromes can be distinguished empirically (i.e., based on separate latent variables) and phenomenologically (i.e., how each defines emotion, identity, and relational dysregulation). The emerging findings from person-centered research suggest that PTSD and DSO often occur in the absence of BPD symptoms, but that BPD often occurs with comorbid PTSD and DSO symptoms than alone. Although there is a sub-set of BPD-diagnosed persons who have no history of childhood maltreatment or other trauma exposure, a larger sub-group of people with BPD have traumatic antecedents beginning in childhood and continuing with re-victimization in adolescence and adulthood.

These findings raise the possibility of the existence of a continuum of post-traumatic syndromes that begins with childhood maltreatment or other traumatic victimization and then progresses with increasing morbidity from PTSD to cPTSD/DSO and ultimately to comorbid BPD/cPTSD. An updated version [10] of the classic general adaptation syndrome [152] and the foundational stress response theory of Horowitz [72], may provide a potential conceptual framework by postulating that stress reactions proceed through four hierarchical phases: freeze, flight, fight, and faint (or tonic immobility). PTSD involves symptoms consistent with a freeze response, including rapid orienting responses to scan the environment for stressors (i.e., hypervigilance) and for ways to avoid harm or signs of potential threat (i.e., avoidance based on intrusive re-experiencing of trauma memories). Although the body appears still, physiologically the freeze response is characterized by hyperarousal consistent with PTSD. By contrast, the DSO symptoms of cPTSD are consistent with the flight response, which is characterized by both unmodulated distress (i.e., DSO’s difficulty in self-calming, guilt, and sense of worthlessness) and both conscious and unconscious attempts to escape from further harm (i.e., DSO’s emotional numbing and relational detachment). Flight responses involve active attempts to restore safety by disengaging from sources of harm (e.g., fear of closeness) and distress, which are highly self-referential (i.e., associated with the DMN) and include attempts to mobilize executive problem solving and decision-making (i.e., associated with the PfC). Thus, cPTSD could be understood as the maladaptive persistence of initially adaptive stress reactions that progress from hypervigilance (i.e., PTSD) to emotional/relational shut-down (i.e., DSO).

BPD could emerge as a fight response when executive control capabilities are not sufficient to sustain PTSD’s freeze/hypervigilance and cPTSD’s flight/detachment, which is consistent with evidence of diminished connectivity within the salience network and the DMN, and hypoactivation of the PfC plus hyperactivation of the amygdala, in BPD. Instead of attempts to cope by means of vigilance or detachment, BPD involves reacting in a fight mode with impulsive, disorganized, and hostile behavior in relationships and limited or no sense of self-awareness and self-efficacy. The fight reaction characterizing BPD includes a surge in bodily arousal initiated by the brain’s innate alarm system [102], and desperate attempts to prevent or retaliate for perceived or real abandonment. The alternating enmeshment in and rejection of relationships characterizing BPD also is consistent with the emotional dysregulation and deficits in executive function that have been found to occur among individuals who are experiencing disorganized attachment.

Dissociation can play a role at any stage in this hypothesized progression from PTSD to cPTSD/DSO to BPD. In PTSD, dissociation takes the form of either flashbacks or the derealization and depersonalization characterizing the dissociative sub-type. In cPTSD/ DSO, dissociation is not a core symptom, but may play a role in extreme emotional numbing and relational detachment. In BPD, extreme dissociative states occur in transient episodes. The common denominator in these diverse manifestations of pathological dissociation is a state of a state of physical or behavioral disorientation and shut-down that may be the result of the final stage of the stress response when freeze, flight, and fight responses have failed to restore safety and homeostasis: tonic immobility [186]. Tonic immobility refers to a shift in the stress response from arousal (mediated by the sympathetic branch of the autonomic nervous system) to extreme deactivation (mediated by the parasympathetic branch) [135]. Pathological dissociation may or may not involve physical and behavioral immobility, it clearly involves a loss of executive control capacities sufficient to result in cognitively and self-referentially immobilized. Tonic immobility has been shown to be prevalent (i.e., in 75% of cases) among adults in treatment for chronic PTSD, many of whom may also have met criteria for cPTSD or BPD [28]. Whether dissociation in PTSD, cPTSD, and BPD represents a stress response of tonic immobility, and how this is similar or differs for these disorders, warrants further research.

It is important to note, however, that there is no evidence as yet, to our knowledge, that directly demonstrates that PTSD, cPTSD, and BPD form a continuum based on either asequential progression over time or a hierarchy of levels of psychopathological severity. Prospective longitudinal research with repeated assessments of the disorders’ symptoms across critical developmental periods and transitions across the lifespan would be necessary to determine whether, and under what conditions, there is temporal sequencing in the onset and progression of the disorders. Confirmatory factor analytic or structural equation model tests of the structure of the disorders’ symptoms would be necessary to determine whether they form an empirically discernable hierarchy. Alternative etiological factors other than exposure to traumatic stressors that may lead to or exacerbate BPD and PTSD (and probably also cPTSD) would have to be assessed (e.g., genetics, temperamental match between child and the primary caregiver(s), family relationships, extra-familial socioeconomic adversities). It also is possible to hypothesize that BPD could develop independently of PTSD or cPTSD, and could then lead to traumatic exposure or a sensitization to the impact of such exposure, that subsequently might lead to the onset of one or both of the traumatic stress disorders. The null hypothesis, that PTSD, cPTSD, and BPD do not fall on a continuum, also could take several forms worthy of investigation. For example, the disorders could be concurrently or sequentially comorbid as a result of etiological factors related to each disorder simply co-occurring by chance or due to unrelated caravans of adversity. By posing the hypothesis that PTSD, cPTSD, and BPD form a continuum we actually are issuing a call to action to clinical researchers and practitioners to examine not only that possibility by also many alternative scenarios that could increase our understanding of the relationship among these disorders.

In sum, the inter-relationships of PTSD, cPTSD/DSO, and BPD are becoming clearer as epidemiological, psychometric, clinical, and neurobiological research has progressed, but conceptual frameworks such as that provided by stress response theory are needed to guide systematic and theory-grounded future research in this crucial mental health domain.

## Data Availability

Not applicable. No original data were collected for this review article.

## References

[1] Aguilar-Ortiz S, Salgado-Pineda P, Vega D, Pascual JC, Marco-Pallares J, Soler J (2020). Evidence for default mode network dysfunction in borderline personality disorder. Psychol Med.

[2] Akiki TJ, Averill CL, Wrocklage KM, Scott JC, Averill LA, Schweinsburg B (2018). Default mode network abnormalities in posttraumatic stress disorder: a novel network-restricted topology approach. Neuroimage.

[3] American Psychiatric Association (2013). Diagnostic and statistical manual of mental disorders.

[4] Armenti NA, Babcock JC. Borderline personality features, anger, and intimate partner violence: an experimental manipulation of rejection. J Interpers Violence. 2018:886260518771686. 10.1177/0886260518771686. 10.1177/0886260518771686 29673305

[5] Beeney JE, Wright AGC, Stepp SD, Hallquist MN, Lazarus SA, Beeney JRS (2017). Disorganized attachment and personality functioning in adults: a latent class analysis. Pers Disord.

[6] Ben-Ezra M, Karatzias T, Hyland P, Brewin CR, Cloitre M, Bisson JI, et al. Posttraumatic stress disorder (PTSD) and complex PTSD (CPTSD) as per ICD-11 proposals: a population study in Israel. Depress Anxiety. 2018. 10.1002/da.22723. 10.1002/da.22723 29451956

[7] Benedict TM, Keenan PG, Nitz AJ, Moeller-Bertram T. Post-traumatic stress disorder symptoms contribute to worse pain and health outcomes in veterans with PTSD compared to those without: a systematic review with meta-analysis. Mil Med. 2020. 10.1093/milmed/usaa052. 10.1093/milmed/usaa052 32248229

[8] Bilek E, Itz ML, Stossel G, Ma R, Berhe O, Clement L (2019). Deficient amygdala habituation to threatening stimuli in borderline personality disorder relates to adverse childhood experiences. Biol Psychiatry.

[9] Bohus M, Kleindienst N, Hahn C, Muller-Engelmann M, Ludascher P, Steil R, et al. Dialectical behavior therapy for posttraumatic stress disorder (DBT-PTSD) compared with cognitive processing therapy (CPT) in complex presentations of PTSD in women survivors of childhood abuse: a randomized clinical trial. JAMA Psychiatry. 2020. 10.1001/jamapsychiatry.2020.2148. 10.1001/jamapsychiatry.2020.2148 PMC7376475 32697288

[10] Bracha HS (2004). Freeze, flight, fight, fright, faint; Adaptationist perspectives on the acute stress response Spectrum. CNS Spectrums.

[11] Breuer F, Greggersen W, Kahl KG, Schweiger U, Westermair AL (2020). Caught in a web of trauma: network analysis of childhood adversity and adult mental ill-health. Child Abuse Negl.

[12] Brewin CR, Cloitre M, Hyland P, Shevlin M, Maercker A, Bryant RA (2017). A review of current evidence regarding the ICD-11 proposals for diagnosing PTSD and complex PTSD. Clin Psychol Rev.

[13] Brumariu LE, Owen MT, Dyer N, Lyons-Ruth K. Developmental pathways to BPD-related features in adolescence: infancy to age 15. J Personal Disord. 2020:1–26. 10.1521/pedi_2020_34_480. 10.1521/pedi_2020_34_480 32539619

[14] Buckholdt KE, Weiss NH, Young J, Gratz KL (2015). Exposure to violence, posttraumatic stress symptoms, and borderline personality pathology among adolescents in residential psychiatric treatment: the influence of emotion Dysregulation. Child Psychiatry Hum Dev.

[15] Byun S, Brumariu LE, Lyons-Ruth K (2016). Disorganized attachment in young adulthood as a partial mediator of relations between severity of childhood abuse and dissociation. J Trauma Dissociation.

[16] Cassidy J (1994). Emotion regulation: influences of attachment relationships. Monogr Soc Res Child Dev.

[17] Chung BY, Hensel S, Schmidinger I, Bekrater-Bodmann R, Flor H (2020). Dissociation proneness and pain hyposensitivity in current and remitted borderline personality disorder. Eur J Pain.

[18] Cloitre M, Garvert DW, Brewin CR, Bryant RA, Maercker A. Evidence for proposed ICD-11 PTSD and complex PTSD: a latent profile analysis. Eur J Psychotraumatol. 2013;4. 10.3402/ejpt.v4i0.20706. 10.3402/ejpt.v4i0.20706 PMC3656217 23687563

[19] Cloitre M, Garvert DW, Weiss B, Carlson EB, Bryant RA. Distinguishing PTSD, complex PTSD, and borderline personality disorder: a latent class analysis. Eur J Psychotraumatol. 2014;5. 10.3402/ejpt.v5.25097. 10.3402/ejpt.v5.25097 PMC4165723 25279111

[20] Cloitre M, Hyland P, Bisson JI, Brewin CR, Roberts NP, Karatzias T, Shevlin M (2019). ICD-11 posttraumatic stress disorder and complex posttraumatic stress disorder in the United States: a population-based study. J Trauma Stress.

[21] Cloitre M, Shevlin M, Brewin CR, Bisson JI, Roberts NP, Maercker A (2018). The international trauma questionnaire: development of a self-report measure of ICD-11 PTSD and complex PTSD. Acta Psychiatr Scand.

[22] Courtois CA, Ford JD, Courtois CA (2020). Therapeutic alliance and risk management. Treatment of complex traumatic stress disorders in adults.

[23] Courtois CA, Ford JD, Cloitre M, Schnyder U, Ford JD, Courtois CA (2020). Best practices in psychotherapy for adults. Treatment of complex traumatic stress disorders in adults.

[24] Cui N, Liu J (2020). Physical abuse, emotional abuse, and neglect and childhood behavior problems: a meta-analysis of studies in mainland China. Trauma Violence Abuse.

[25] D'Andrea W, Ford JD, Stolbach B, Spinazzola J, van der Kolk BA (2012). Understanding interpersonal trauma in children: why we need a developmentally appropriate trauma diagnosis. Am J Orthop.

[26] Davis JP, Dworkin ER, Helton J, Prindle J, Patel S, Dumas TM, Miller S (2019). Extending poly-victimization theory: differential effects of adolescents’ experiences of victimization on substance use disorder diagnoses upon treatment entry. Child Abuse Negl.

[27] de Aquino Ferreira LF, Queiroz Pereira FH, Neri Benevides AML, Aguiar Melo MC (2018). Borderline personality disorder and sexual abuse: a systematic review. Psychiatry Res.

[28] de Kleine RA, Hagenaars MA, van Minnen A (2018). Tonic immobility during re-experiencing the traumatic event in posttraumatic stress disorder. Psychiatry Res.

[29] Del Rio-Casanova L, Gonzalez A, Paramo M, Van Dijke A, Brenlla J. Emotion regulation strategies in trauma-related disorders: pathways linking neurobiology and clinical manifestations. Rev Neurosci. 2016. 10.1515/revneuro-2015-0045. 10.1515/revneuro-2015-0045 26812780

[30] Doell KC, Olie E, Courtet P, Corradi-Dell'Acqua C, Perroud N, Schwartz S (2020). Atypical processing of social anticipation and feedback in borderline personality disorder. Neuroimage Clin.

[31] Doering S (2019). Borderline personality disorder in patients with medical illness: a review of assessment, prevalence, and treatment options. Psychosom Med.

[32] Dorahy MJ, Corry M, Black R, Matheson L, Coles H, Curran D (2017). Shame, dissociation, and complex PTSD symptoms in traumatized psychiatric and control groups: direct and indirect associations with relationship distress. J Clin Psychol.

[33] Dorahy MJ, Corry M, Shannon M, Webb K, McDermott B, Ryan M, Dyer KF (2013). Complex trauma and intimate relationships: the impact of shame, guilt and dissociation. J Affect Disord.

[34] Dunn NJ, Yanasak E, Schillaci J, Simotas S, Rehm LP, Souchek J (2004). Personality disorders in veterans with posttraumatic stress disorder and depression. J Trauma Stress.

[35] Elklit A, Hyland P, Shevlin M. Evidence of symptom profiles consistent with posttraumatic stress disorder and complex posttraumatic stress disorder in different trauma samples. Eur J Psychotraumatol. 2014;5. 10.3402/ejpt.v5.24221. 10.3402/ejpt.v5.24221 PMC4028608 24851144

[36] Finkelhor D, Turner HA, Shattuck A, Hamby SL (2015). Prevalence of childhood exposure to violence, crime, and abuse: results from the National Survey of Children's exposure to violence. JAMA Pediatr.

[37] Finkelhor D, Vanderminden J, Turner H, Hamby S, Shattuck A (2014). Child maltreatment rates assessed in a national household survey of caregivers and youth. Child Abuse Negl.

[38] Fishbain DA, Pulikal A, Lewis JE, Gao J (2017). Chronic pain types differ in their reported prevalence of post -traumatic stress disorder (PTSD) and there is consistent evidence that chronic pain is associated with PTSD: an evidence-based structured systematic review. Pain Med.

[39] Ford JD (2019). Commentary on the special section on complex PTSD: still going strong after all these years. J Trauma Stress.

[40] Ford JD, Ford JD, Courtois CA (2020). Developmental neurobiology. Treatment of complex traumatic stress disorders in adults.

[41] Ford JD (2020). New findings questioning the construct validity of complex posttraumatic stress disorder (cPTSD): let's take a closer look. Eur J Psychotraumatol.

[42] Ford JD, Charak R, Modrowski CA, Kerig PK (2018). PTSD and dissociation symptoms as mediators of the relationship between polyvictimization and psychosocial and behavioral problems among justice-involved adolescents. J Trauma Dissociation.

[43] Ford JD, Courtois CA. Complex PTSD, affect dysregulation, and borderline personality disorder. Borderline Personal Disord Emot Dysregul, 2014;1:9. 10.1186/2051-6673-1-9 10.1186/2051-6673-1-9 PMC4579513 26401293

[44] Ford JD, Courtois CA (2020). Treatment of complex traumatic stress disorders in adults.

[45] Ford JD, Grasso D, Greene C, Levine J, Spinazzola J, van der Kolk B (2013). Clinical significance of a proposed developmental trauma disorder diagnosis: results of an international survey of clinicians. J Clin Psychiatry.

[46] Ford JD, Spinazzola J, van der Kolk B, Grasso D (2018). Toward an empirically-based developmental trauma disorder diagnosis for children: factor structure, item characteristics, reliability, and validity of the developmental trauma disorder semi-structured interview (DTD-SI). J Clin Psychiatry.

[47] Fossati A, Gratz KL, Somma A, Maffei C, Borroni S (2016). The mediating role of emotion Dysregulation in the relations between childhood trauma history and adult attachment and borderline personality disorder features: a study of Italian nonclinical participants. J Personal Disord.

[48] Foxhall M, Hamilton-Giachritsis C, Button K (2019). The link between rejection sensitivity and borderline personality disorder: a systematic review and meta-analysis. Br J Clin Psychol.

[49] Frankenburg FR, Fitzmaurice GM, Zanarini MC. The use of prescription opioid medication by patients with borderline personality disorder and axis II comparison subjects: a 10-year follow-up study. J Clin Psychiatry. 2014. 10.4088/JCP.13m08557. 10.4088/JCP.13m08557 PMC4019694 24500123

[50] Frias A, Palma C (2015). Comorbidity between post-traumatic stress disorder and borderline personality disorder: a review. Psychopathology.

[51] Frost R, Hyland P, McCarthy A, Halpin R, Shevlin M, Murphy J (2019). The complexity of trauma exposure and response: profiling PTSD and CPTSD among a refugee sample. Psychol Trauma.

[52] Frost R, Hyland P, Shevlin M, Murphy J. Distinguishing complex PTSD from Borderline Personality Disorder among individuals with a history of sexual_trauma: A atent class analysis. Eur J Trauma Dissociation. 2020;4(1):100080. 10.1016/j.ejtd.2018.08.004.

[53] Gardner AA, Zimmer-Gembeck MJ, Campbell SM (2020). Attachment and emotion regulation: a person-centred examination and relations with coping with rejection, friendship closeness, and emotional adjustment. Br J Dev Psychol.

[54] Gilbar O, Hyland P, Cloitre M, Dekel R (2018). ICD-11 complex PTSD among Israeli male perpetrators of intimate partner violence: construct validity and risk factors. J Anxiety Disord.

[55] Girme YU, Jones RE, Fleck C, Simpson JA, Overall NC. Infants’ attachment insecurity predicts attachment-relevant emotion regulation strategies in adulthood. Emotion. 2020. 10.1037/emo0000721. 10.1037/emo0000721 PMC7343591 31916790

[56] Gonzalez RA, Igoumenou A, Kallis C, Coid JW (2016). Borderline personality disorder and violence in the UK population: categorical and dimensional trait assessment. BMC Psychiatry.

[57] Grasso DJ, Dierkhising CB, Branson CE, Ford JD, Lee R (2016). Developmental patterns of adverse childhood experiences and current symptoms and impairment in youth referred for trauma-specific services. J Abnorm Child Psychol.

[58] Greene T, Neria Y, Gross R (2016). Prevalence, detection and correlates of PTSD in the primary care setting: a systematic review. J Clin Psychol Med Settings.

[59] Gunderson JG, Chu JA (1993). Treatment implications of past trauma in borderline personality disorder. Harv Rev Psychiatry.

[60] Hamby S, Finkelhor D, Turner H, Ormrod R (2010). The overlap of witnessing partner violence with child maltreatment and other victimizations in a nationally representative survey of youth. Child Abuse Negl.

[61] Harford TC, Chen CM, Kerridge BT, Grant BF (2019). Borderline personality disorder and violence toward self and others: a National Study. J Pers Disord.

[62] Harned MS, Gallop RJ, Valenstein-Mah HR (2018). What changes when? The course of improvement during a stage-based treatment for suicidal and self-injuring women with borderline personality disorder and PTSD. Psychother Res.

[63] Harned MS, Korslund KE, Linehan MM (2014). A pilot randomized controlled trial of dialectical behavior therapy with and without the dialectical behavior therapy prolonged exposure protocol for suicidal and self-injuring women with borderline personality disorder and PTSD. Behav Res Ther.

[64] Harned MS, Wilks CR, Schmidt SC, Coyle TN (2018). Improving functional outcomes in women with borderline personality disorder and PTSD by changing PTSD severity and post-traumatic cognitions. Behav Res Ther.

[65] Harricharan S, Nicholson AA, Thome J, Densmore M, McKinnon MC, Theberge J (2020). PTSD and its dissociative subtype through the lens of the insula: anterior and posterior insula resting-state functional connectivity and its predictive validity using machine learning. Psychophysiology.

[66] Haselgruber A, Solva K, Lueger-Schuster B (2020). Validation of ICD-11 PTSD and complex PTSD in foster children using the international trauma questionnaire. Acta Psychiatr Scand.

[67] Hebert M, Langevin R, Charest F (2020). Disorganized attachment and emotion dysregulation as mediators of the association between sexual abuse and dissociation in preschoolers. J Affect Disord.

[68] Hebert M, Langevin R, Oussaid E (2018). Cumulative childhood trauma, emotion regulation, dissociation, and behavior problems in school-aged sexual abuse victims. J Affect Disord.

[69] Henschel S, Doba K, Nandrino JL (2019). Emotion regulation processes and Psychoform and somatoform dissociation in adolescents and Young adults with cumulative maltreatment. J Trauma Dissociation.

[70] Herman JL (1992). Complex PTSD: a syndrome in survivors of prolonged and repeated trauma. J Trauma Stress.

[71] Herzog JI, Niedtfeld I, Rausch S, Thome J, Mueller-Engelmann M, Steil R (2019). Increased recruitment of cognitive control in the presence of traumatic stimuli in complex PTSD. Eur Arch Psychiatry Clin Neurosci.

[72] Horowitz M (1986). Stress response syndromes: a review of posttraumatic and adjustment disorders. Hosp Community Psychiatry.

[73] Hyland P, Karatzias T, Shevlin M, Cloitre M (2019). Examining the discriminant validity of complex posttraumatic stress disorder and borderline personality disorder symptoms: results from a United Kingdom population sample. J Trauma Stress.

[74] Hyland P, Karatzias T, Shevlin M, Cloitre M, Ben-Ezra M (2020). A longitudinal study of ICD-11 PTSD and complex PTSD in the general population of Israel. Psychiatry Res.

[75] Hyland P, Shevlin M, Brewin CR, Cloitre M, Downes AJ, Jumbe S (2017). Validation of post-traumatic stress disorder (PTSD) and complex PTSD using the international trauma questionnaire. Acta Psychiatr Scand.

[76] Hyland P, Shevlin M, Fyvie C, Cloitre M, Karatzias T (2020). The relationship between ICD-11 PTSD, complex PTSD and dissociative experiences. J Trauma Dissociation.

[77] Hyland P, Shevlin M, Fyvie C, Karatzias T (2018). Posttraumatic stress disorder and complex posttraumatic stress disorder in DSM-5 and ICD-11: clinical and behavioral correlates. J Trauma Stress.

[78] Hyland P, Vallieres F, Cloitre M, Ben-Ezra M, Karatzias T, Olff M, et al. Trauma, PTSD, and complex PTSD in the Republic of Ireland: prevalence, service use, comorbidity, and risk factors. Soc Psychiatry Psychiatr Epidemiol. 2020. 10.1007/s00127-020-01912-x. 10.1007/s00127-020-01912-x 32632598

[79] Jackson MA, Sippel LM, Mota N, Whalen D, Schumacher JA (2015). Borderline personality disorder and related constructs as risk factors for intimate partner violence perpetration. Aggress Violent Behav.

[80] Jakovina T, Crnkovic Batista M, RaZic Pavicic A, Zuric Jakovina I, Begovac I (2018). Emotional dysregulation and attachment dimensions in female patients with bulimia nervosa. Psychiatry.

[81] Jimenez XF (2013). Patients with borderline personality disorder who are chronically suicidal: therapeutic alliance and therapeutic limits. Am J Psychother.

[82] Johnson BN, Lumley MA, Cheavens JS, McKernan LC (2020). Exploring the links among borderline personality disorder symptoms, trauma, and pain in patients with chronic pain disorders. J Psychosom Res.

[83] Jowett S, Karatzias T, Albert I (2020). Multiple and interpersonal trauma are risk factors for both post-traumatic stress disorder and borderline personality disorder: a systematic review on the traumatic backgrounds and clinical characteristics of comorbid post-traumatic stress disorder/borderline personality disorder groups versus single-disorder groups. Psychol Psychother.

[84] Jowett S, Karatzias T, Shevlin M, Albert I (2020). Differentiating symptom profiles of ICD-11 PTSD, complex PTSD, and borderline personality disorder: a latent class analysis in a multiply traumatized sample. Pers Disord.

[85] Karatzias T, Cloitre M (2019). Treating adults with complex posttraumatic stress disorder using a modular approach to treatment: rationale, evidence, and directions for future research. J Trauma Stress.

[86] Karatzias T, Murphy P, Cloitre M, Bisson J, Roberts N, Shevlin M (2019). Psychological interventions for ICD-11 complex PTSD symptoms: systematic review and meta-analysis. Psychol Med.

[87] Karatzias T, Shevlin M, Fyvie C, Hyland P, Efthymiadou E, Wilson D (2016). An initial psychometric assessment of an ICD-11 based measure of PTSD and complex PTSD (ICD-TQ): evidence of construct validity. J Anxiety Disord.

[88] Karatzias T, Shevlin M, Hyland P, Brewin CR, Cloitre M, Bradley A (2018). The role of negative cognitions, emotion regulation strategies, and attachment style in complex post-traumatic stress disorder: implications for new and existing therapies. Br J Clin Psychol.

[89] Kerig PK, Modrowski CA (2018). Testing gender-differentiated models of the mechanisms linking polyvictimization and youth offending: numbing and callousness versus dissociation and borderline traits. J Trauma Dissociation.

[90] Kessler RC, Aguilar-Gaxiola S, Alonso J, Benjet C, Bromet EJ, Cardoso G, et al. Trauma and PTSD in the WHO world mental health surveys. Eur J Psychotraumatol, 8. 2017;(sup5):1353383. 10.1080/20008198.2017.1353383. 10.1080/20008198.2017.1353383 PMC5632781 29075426

[91] Khosravi M (2020). Child maltreatment-related dissociation and its core mediation schemas in patients with borderline personality disorder. BMC Psychiatry.

[92] Khoury JE, Zona K, Bertha E, Choi-Kain L, Hennighausen K, Lyons-Ruth K. Disorganized attachment interactions among Young adults with borderline personality disorder, other diagnoses, and no diagnosis. J Personal Disord. 2019:1–21. 10.1521/pedi_2019_33_408. 10.1521/pedi_2019_33_408 30785838

[93] Kind S, Otis JD (2019). The interaction between chronic pain and PTSD. Curr Pain Headache Rep.

[94] Knefel M, Lueger-Schuster B, Karatzias T, Shevlin M, Hyland P (2019). From child maltreatment to ICD-11 complex post-traumatic stress symptoms: the role of emotion regulation and re-victimisation. J Clin Psychol.

[95] Knefel M, Tran US, Lueger-Schuster B (2016). The association of posttraumatic stress disorder, complex posttraumatic stress disorder, and borderline personality disorder from a network analytical perspective. J Anxiety Disord.

[96] Krause-Utz A, Mertens LJ, Renn JB, Lucke P, Wohlke AZ, van Schie CC, et al. Childhood maltreatment, borderline personality features, and coping as predictors of intimate partner violence. J Interpers Violence. 2018:886260518817782. 10.1177/0886260518817782. 10.1177/0886260518817782 PMC8202213 30596325

[97] Krause-Utz A, Winter D, Niedtfeld I, Schmahl C (2014). The latest neuroimaging findings in borderline personality disorder. Curr Psychiatry Rep.

[98] Krause-Utz A, Winter D, Schriner F, Chiu CD, Lis S, Spinhoven P (2018). Reduced amygdala reactivity and impaired working memory during dissociation in borderline personality disorder. Eur Arch Psychiatry Clin Neurosci.

[99] Kulkarni J (2017). Complex PTSD - a better description for borderline personality disorder?. Australassian Psychiatry.

[100] Lanius RA (2015). Trauma-related dissociation and altered states of consciousness: a call for clinical, treatment, and neuroscience research. Eur J Psychotraumatol.

[101] Lanius RA, Brand B, Vermetten E, Frewen PA, Spiegel D (2012). The dissociative subtype of posttraumatic stress disorder: rationale, clinical and neurobiological evidence, and implications. Depress Anxiety.

[102] Lanius RA, Rabellino D, Boyd JE, Harricharan S, Frewen PA, McKinnon MC (2017). The innate alarm system in PTSD: conscious and subconscious processing of threat. Curr Opin Psychol.

[103] Lebois LAM, Li M, Baker JT, Wolff JD, Wang D, Lambros AM, et al. Large-scale functional brain network architecture changes associated with trauma-related dissociation. Am J Psychiatr. 2020:appiajp202019060647. 10.1176/appi.ajp.2020.19060647. 10.1176/appi.ajp.2020.19060647 PMC8030225 32972201

[104] Liese BS, Kim HS, Hodgins DC (2020). Insecure attachment and addiction: testing the mediating role of emotion dysregulation in four potentially addictive behaviors. Addict Behav.

[105] Linehan MM (1987). Dialectical behavior therapy for borderline personality disorder. Theory and method. Bull Menn Clin.

[106] Linehan MM, Korslund KE, Harned MS, Gallop RJ, Lungu A, Neacsiu AD, et al. Dialectical behavior therapy for high suicide risk in individuals with borderline personality disorder: a randomized clinical trial and component analysis. JAMA Psychiatry. 2015. 10.1001/jamapsychiatry.2014.3039. 10.1001/jamapsychiatry.2014.3039 25806661

[107] Liu N, Zhang Y, Brady HJ, Cao Y, He Y, Zhang Y (2012). Relation between childhood maltreatment and severe intrafamilial male-perpetrated physical violence in Chinese community: the mediating role of borderline and antisocial personality disorder features. Aggress Behav.

[108] Luyten P, Campbell C, Fonagy P (2020). Borderline personality disorder, complex trauma, and problems with self and identity: a social-communicative approach. J Pers.

[109] Lyons-Ruth K, Brumariu LE, Bureau JF, Hennighausen K, Holmes B (2015). Role confusion and disorientation in Young adult-parent interaction among individuals with borderline symptomatology. J Personal Disord.

[110] Lyons-Ruth K, Bureau JF, Holmes B, Easterbrooks A, Brooks NH (2013). Borderline symptoms and suicidality/self-injury in late adolescence: prospectively observed relationship correlates in infancy and childhood. Psychiatry Res.

[111] Lyons-Ruth K, Pechtel P, Yoon SA, Anderson CM, Teicher MH (2016). Disorganized attachment in infancy predicts greater amygdala volume in adulthood. Behav Brain Res.

[112] Lyons-Ruth K, Spielman E (2004). Disorganized infant attachment strategies and helpless-fearful profiles of parenting: integrating attachment research with clinical intervention. Infant Ment Health J.

[113] Maercker A, Hecker T, Augsburger M, Kliem S (2018). ICD-11 prevalence rates of posttraumatic stress disorder and complex posttraumatic stress disorder in a German Nationwide sample. J Nerv Ment Dis.

[114] Malik S, Wells A, Wittkowski A (2015). Emotion regulation as a mediator in the relationship between attachment and depressive symptomatology: a systematic review. J Affect Disord.

[115] Marcusson-Clavertz D, Gusic S, Bengtsson H, Jacobsen H, Cardena E (2017). The relation of dissociation and mind wandering to unresolved/disorganized attachment: an experience sampling study. Attach Hum Dev.

[116] Miljkovitch R, Deborde AS, Bernier A, Corcos M, Speranza M, Pham-Scottez A (2018). Borderline personality disorder in adolescence as a generalization of disorganized attachment. Front Psychol.

[117] Moller L, Augsburger M, Elklit A, Sogaard U, Simonsen E (2020). Traumatic experiences, ICD-11 PTSD, ICD-11 complex PTSD, and the overlap with ICD-10 diagnoses. Acta Psychiatr Scand.

[118] Moore KE, Gobin RL, McCauley HL, Kao CW, Anthony SM, Kubiak S (2018). The relation of borderline personality disorder to aggression, victimization, and institutional misconduct among prisoners. Compr Psychiatry.

[119] Mosquera D, Gonzalez A, Leeds AM (2014). Early experience, structural dissociation, and emotional dysregulation in borderline personality disorder: the role of insecure and disorganized attachment. Borderline Pers Disord Emot Dysregul.

[120] Munjiza J, Britvic D, Crawford MJ (2019). Lasting personality pathology following exposure to severe trauma in adulthood: retrospective cohort study. BMC Psychiatry.

[121] Munro OE, Sellbom M (2020). Elucidating the relationship between borderline personality disorder and intimate partner violence. Personal Ment Health.

[122] Murphy S, Elklit A, Dokkedahl S, Shevlin M (2016). Testing the validity of the proposed ICD-11 PTSD and complex PTSD criteria using a sample from northern Uganda. Eur J Psychotraumatol.

[123] Murphy S, Elklit A, Murphy J, Hyland P, Shevlin M (2017). A cross-lagged panel study of dissociation and posttraumatic stress in a treatment-seeking sample of survivors of childhood sexual abuse. J Clin Psychol.

[124] Myrick AC, Webermann AR, Loewenstein RJ, Lanius R, Putnam FW, Brand BL (2017). Six-year follow-up of the treatment of patients with dissociative disorders study. Eur J Psychotraumatol.

[125] Neacsiu AD, Lungu A, Harned MS, Rizvi SL, Linehan MM (2014). Impact of dialectical behavior therapy versus community treatment by experts on emotional experience, expression, and acceptance in borderline personality disorder. Behav Res Ther.

[126] Newhill CE, Eack SM, Mulvey EP (2012). A growth curve analysis of emotion dysregulation as a mediator for violence in individuals with and without borderline personality disorder. J Personal Disord.

[127] Niedtfeld I, Renkewitz F, Madebach A, Hillmann K, Kleindienst N, Schmahl C, Schulze L (2020). Enhanced memory for negative social information in borderline personality disorder. J Abnorm Psychol.

[128] Niedtfeld I, Schulze L, Krause-Utz A, Demirakca T, Bohus M, Schmahl C (2013). Voxel-based morphometry in women with borderline personality disorder with and without comorbid posttraumatic stress disorder. Plos One.

[129] Norman RE, Byambaa M, De R, Butchart A, Scott J, Vos T (2012). The long-term health consequences of child physical abuse, emotional abuse, and neglect: a systematic review and meta-analysis. Plos Med.

[130] Peng W, Liu Z, Liu Q, Chu J, Zheng K, Wang J, et al. Insecure attachment and maladaptive emotion regulation mediating the relationship between childhood trauma and borderline personality features. Depress Anxiety. 2020. 10.1002/da.23082. 10.1002/da.23082 32720464

[131] Perkonigg A, Hofler M, Cloitre M, Wittchen HU, Trautmann S, Maercker A (2016). Evidence for two different ICD-11 posttraumatic stress disorders in a community sample of adolescents and young adults. Eur Arch Psychiatry Clin Neurosci.

[132] Peters JR, Derefinko KJ, Lynam DR (2017). Negative urgency accounts for the association between borderline personality features and intimate partner violence in Young men. J Personal Disord.

[133] Petrowski K, Wintermann GB, Hubner T, Smolka MN, Donix M (2019). Neural responses to faces of attachment figures and unfamiliar faces: associations with organized and disorganized attachment representations. J Nerv Ment Dis.

[134] Popkirov S, Flasbeck V, Schlegel U, Juckel G, Brune M (2019). Childhood trauma and dissociative symptoms predict frontal EEG asymmetry in borderline personality disorder. J Trauma Dissociation.

[135] Porges SW (2009). The polyvagal theory: new insights into adaptive reactions of the autonomic nervous system. Cleve Clin Med J.

[136] Porter C, Palmier-Claus J, Branitsky A, Mansell W, Warwick H, Varese F (2020). Childhood adversity and borderline personality disorder: a meta-analysis. Acta Psychiatr Scand.

[137] Quattrini G, Pini L, Pievani M, Magni LR, Lanfredi M, Ferrari C (2019). Abnormalities in functional connectivity in borderline personality disorder: correlations with metacognition and emotion dysregulation. Psychiatry Res Neuroimaging.

[138] Quek J, Newman LK, Bennett C, Gordon MS, Saeedi N, Melvin GA (2017). Reflective function mediates the relationship between emotional maltreatment and borderline pathology in adolescents: a preliminary investigation. Child Abuse Negl.

[139] Reyes-Ortega MA, Miranda EM, Fresan A, Vargas AN, Barragan SC, Robles Garcia R, Arango I (2020). Clinical efficacy of a combined acceptance and commitment therapy, dialectical behavioural therapy, and functional analytic psychotherapy intervention in patients with borderline personality disorder. Psychol Psychother.

[140] Riem MME, van Hoof MJ, Garrett AS, Rombouts S, van der Wee NJA, van, I. M. H., & Vermeiren, R. (2019). General psychopathology factor and unresolved-disorganized attachment uniquely correlated to white matter integrity using diffusion tensor imaging. Behav Brain Res.

[141] Rometsch-Ogioun El Sount C, Windthorst P, Denkinger J, Ziser K, Nikendei C, Kindermann D (2019). Chronic pain in refugees with posttraumatic stress disorder (PTSD): a systematic review on patients’ characteristics and specific interventions. J Psychosom Res.

[142] Roth S, Newman E, Pelcovitz D, vander Kolk B, Mandel FS (1997). Complex PTSD in victims exposed to sexual and physical abuse: Results from the DSM-IV field trial for posttraumatic stress disorder. J Trauma Stress.

[143] Sachser C, Keller F, Goldbeck L (2017). Complex PTSD as proposed for ICD-11: validation of a new disorder in children and adolescents and their response to trauma-focused cognitive behavioral therapy. J Child Psychol Psychiatry.

[144] Salgado RM, Pedrosa R, Bastos-Leite AJ (2020). Dysfunction of empathy and related processes in borderline personality disorder: a systematic review. Harv Rev Psychiatry.

[145] Sbarra DA, Hazan C (2008). Coregulation, dysregulation, self-regulation: an integrative analysis and empirical agenda for understanding adult attachment, separation, loss, and recovery. Personal Soc Psychol Rev.

[146] Scalabrini A, Cavicchioli M, Fossati A, Maffei C (2017). The extent of dissociation in borderline personality disorder: a meta-analytic review. J Trauma Dissociation.

[147] Schaefer C, Enning F, Mueller JK, Bumb JM, Rohleder C, Odorfer TM (2014). Fatty acid ethanolamide levels are altered in borderline personality and complex posttraumatic stress disorders. Eur Arch Psychiatry Clin Neurosci.

[148] Scheiderer EM, Wood PK, Trull TJ (2015). The comorbidity of borderline personality disorder and posttraumatic stress disorder: revisiting the prevalence and associations in a general population sample. Borderline Pers Disord Emotion Dysregulation.

[149] Scherpiet S, Bruhl AB, Opialla S, Roth L, Jancke L, Herwig U (2014). Altered emotion processing circuits during the anticipation of emotional stimuli in women with borderline personality disorder. Eur Arch Psychiatry Clin Neurosci.

[150] Schmahl C, Meinzer M, Zeuch A, Fichter M, Cebulla M, Kleindienst N (2010). Pain sensitivity is reduced in borderline personality disorder, but not in posttraumatic stress disorder and bulimia nervosa. World J Biol Psychiatry.

[151] Scoboria A, Ford J, Lin HJ, Frisman L (2008). Exploratory and confirmatory factor analyses of the structured interview for disorders of extreme stress. Assessment.

[152] Selye H (1950). Stress and the general adaptation syndrome. Br Med J.

[153] Schulze L, Schulze A, Renneberg B, Schmahl C, Niedtfeld I. Neural Correlates of Affective Disturbances: A Comparative Metaanalysis of Negative Affect Processing in Borderline Personality Disorder, Major Depressive Disorder, and Posttraumatic Stress Disorder. Biol Psychiatry Cogn Neurosci Neuroimaging. 2019;4(3):220–32. 10.1016/j.bpsc.2018.11.004. 10.1016/j.bpsc.2018.11.004 30581154

[154] Sharp C, Vanwoerden S, Jouriles EN, Godfrey DA, Babcock J, McLaren V (2020). Exposure to interparental intimate partner violence and the development of borderline features in adolescents. Child Abuse Negl.

[155] Shevlin M, Hyland P, Karatzias T, Fyvie C, Roberts N, Bisson JI (2017). Alternative models of disorders of traumatic stress based on the new ICD-11 proposals. Acta Psychiatr Scand.

[156] Sinai C, Hirvikoski T, Wiklander M, Nordstrom AL, Nordstrom P, Nilsonne A (2018). Exposure to interpersonal violence and risk of post-traumatic stress disorder among women with borderline personality disorder. Psychiatry Res.

[157] Snoek A, Beekman ATF, Dekker J, Aarts I, van Grootheest G, Blankers M (2020). A randomized controlled trial comparing the clinical efficacy and cost-effectiveness of eye movement desensitization and reprocessing (EMDR) and integrated EMDR-Dialectical Behavioural Therapy (DBT) in the treatment of patients with post-traumatic stress disorder and comorbid (Sub) clinical borderline personality disorder: study design. BMC Psychiatry.

[158] Spinazzola J, Briere J, Ford JD, Courtois CA (2020). Evidence-based psychological assessment for the sequelae of complex trauma. Treatment of complex traumatic stress disorders in adults.

[159] Spinazzola J, Hodgdon H, Liang L, Ford JD, Layne C, Pynoos R (2014). Unseen wounds: the contribution of psychological maltreatment to child and adolescent mental health and risk outcomes. Psychol Trauma Theory Res Pract Policy.

[160] Spinazzola J, van der Kolk B, Ford JD (2018). When nowhere is safe: Trauma history antecedents of Posttraumatic Stress Disorder and Developmental Trauma Disorder in childhood. J Trauma Stress.

[161] Steele K, van der Hart O, Ford JD, Courtois CA (2020). Assessing and treating complex dissociative disorders. Treating complex rraumatic stress disorders in adults 2nd Edition.

[162] Stein DJ, McLaughlin KA, Koenen KC, Atwoli L, Friedman MJ, Hill ED (2014). DSM-5 and ICD-11 definitions of posttraumatic stress disorder: investigating “narrow” and “broad” approaches. Depress Anxiety.

[163] Storebo OJ, Stoffers-Winterling JM, Vollm BA, Kongerslev MT, Mattivi JT, Jorgensen MS (2020). Psychological therapies for people with borderline personality disorder. Cochrane Database Syst Rev.

[164] Tatnell R, Hasking P, Newman L, Taffe J, Martin G (2017). Attachment, emotion regulation, childhood abuse and assault: examining predictors of NSSI among adolescents. Arch Suicide Res.

[165] Teicher MH, Samson JA. Annual Research Review: Enduring neurobiological effects of childhood abuse and neglect. J Child Psychol Psych Allied Discipl. 2016;57(3):241–66. 10.1111/jcpp.12507. 10.1111/jcpp.12507 PMC4760853 26831814

[166] Terpou BA, Harricharan S, McKinnon MC, Frewen P, Jetly R, Lanius RA (2019). The effects of trauma on brain and body: a unifying role for the midbrain periaqueductal gray. J Neurosci Res.

[167] Theoret V, Lapierre A, Blais M, Hebert M. Can emotion Dysregulation explain the association between attachment insecurities and teen dating violence perpetration? J Interpers Violence. 2020:886260520915547. 10.1177/0886260520915547. 10.1177/0886260520915547 32306831

[168] Thomaes K, Dorrepaal E, Draijer N, de Ruiter MB, Elzinga BM, Sjoerds Z (2013). Increased anterior cingulate cortex and hippocampus activation in complex PTSD during encoding of negative words. Soc Cogn Affect Neurosci.

[169] Thomaes K, Dorrepaal E, Draijer NP, de Ruiter MB, Elzinga BM, van Balkom AJ (2009). Increased activation of the left hippocampus region in complex PTSD during encoding and recognition of emotional words: a pilot study. Psychiatry Res.

[170] Thomsen MS, Ruocco AC, Carcone D, Mathiesen BB, Simonsen E (2017). Neurocognitive deficits in borderline personality disorder: associations with childhood trauma and dimensions of personality psychopathology. J Personal Disord.

[171] Turner HA, Shattuck A, Finkelhor D, Hamby S. Effects of poly-victimization on adolescent social support, self-concept, and psychological distress. J Interpers Violence. 2015. 10.1177/0886260515586376. 10.1177/0886260515586376 26033616

[172] Turner HA, Shattuck A, Finkelhor D, Hamby S (2016). Polyvictimization and youth violence exposure across contexts. J Adolesc Health.

[173] Turniansky H, Ben-Dor D, Krivoy A, Weizman A, Shoval G (2019). A history of prolonged childhood sexual abuse is associated with more severe clinical presentation of borderline personality disorder in adolescent female inpatients - a naturalistic study. Child Abuse Negl.

[174] van der Kolk B, Ford JD, Spinazzola J (2019). Comorbidity of developmental trauma disorder (DTD) and post-traumatic stress disorder: findings from the DTD field trial. Eur J Psychotraumatol.

[175] Van Dijke A, Ford JD, Frank L, Van Son M, Van der Hart O (2013). Association of childhood trauma-by-primary-caregiver and affect dysregulation with borderline personality disorder symptoms in adulthood. Psychol Trauma Theory Res Pract Policy.

[176] Van Dijke A, Ford JD, Frank LE, van der Hart O (2015). Association of childhood complex trauma and dissociation with complex posttraumatic stress disorder symptoms in adulthood. J Trauma Dissociation.

[177] Van Dijke A, Ford JD, van der Hart O, van Son M, van der Heijden P, Buhring M (2010). Affect dysregulation in borderline personality disorder and somatoform disorder: differentiating under- and over-regulation. J Personal Disord.

[178] Van Dijke A, Ford JD, Van der Hart O, Van Son M, van der Heijden P, Bühring M (2012). Complex posttraumatic stress disorder in patients with borderline personality disorder and somatoform disorders. Psychol Trauma Theory Res Pract Policy.

[179] Van Dijke A, Hopman JAB, Ford JD (2018). Affect dysregulation, adult attachment problems, and dissociation mediate the relationship between childhood trauma and borderline personality disorder symptoms in adulthood. Eur J Trauma Dissociation.

[180] Van Dijke A, Hopman JAB, Ford JD (2018). Affect dysregulation, psychoform dissociation, and adult relational fears mediate the relationship between childhood trauma and complex posttraumatic stress disorder independent of the symptoms of borderline personality disorder. Eur J Psychotraumatol.

[181] van Hoof MJ, Riem MME, Garrett AS, van der Wee NJA, van IJzendoorn MH, Vermeiren R (2019). Unresolved-disorganized attachment adjusted for a general psychopathology factor associated with atypical amygdala resting-state functional connectivity. Eur J Psychotraumatol.

[182] Vanwoerden S, Leavitt J, Gallagher MW, Temple JR, Sharp C (2019). Dating violence victimization and borderline personality pathology: temporal associations from late adolescence to early adulthood. Pers Disord.

[183] Vermetten E, Spiegel D. Trauma and dissociation: implications for borderline personality disorder. Curr Psych Reports. 2014;16(2):434. 10.1007/s11920-013-0434-8. 10.1007/s11920-013-0434-8 24442670

[184] Villalta L, Khadr S, Chua KC, Kramer T, Clarke V, Viner RM (2020). Complex post-traumatic stress symptoms in female adolescents: the role of emotion dysregulation in impairment and trauma exposure after an acute sexual assault. Eur J Psychotraumatol.

[185] Vogt KS, Norman P (2019). Is mentalization-based therapy effective in treating the symptoms of borderline personality disorder? A systematic review. Psychol Psychother.

[186] Volchan E, Souza GG, Franklin CM, Norte CE, Rocha-Rego V, Oliveira JM (2011). Is there tonic immobility in humans? Biological evidence from victims of traumatic stress. Biol Psychol.

[187] Winsper C, Bilgin A, Wolke D (2020). Associations between infant and toddler regulatory problems, childhood co-developing internalising and externalising trajectories, and adolescent depression, psychotic and borderline personality disorder symptoms. J Child Psychol Psychiatry.

[188] Winter D (2016). Attention to emotional stimuli in borderline personality disorder - a review of the influence of dissociation, self-reference, and psychotherapeutic interventions. Borderline Pers Disord Emot Dysregul.

[189] Wolf EJ, Lunney CA, Miller MW, Resick PA, Friedman MJ, Schnurr PP (2012). The dissociative subtype of PTSD: a replication and extension. Depress Anxiety.

[190] Wolf EJ, Miller MW, Kilpatrick D, Resnick HS, Badour CL, Marx BP (2015). ICD-11 complex PTSD in US national and veteran samples: prevalence and structural associations with PTSD. Clin Psychol Sci.

[191] Zanarini MC, Frankenburg FR, Ridolfi ME, Jager-Hyman S, Hennen J, Gunderson JG (2006). Reported childhood onset of self-mutilation among borderline patients. J Personal Disord.

[192] Zanarini MC, Horz S, Frankenburg FR, Weingeroff J, Reich DB, Fitzmaurice G (2011). The 10-year course of PTSD in borderline patients and axis II comparison subjects. Acta Psychiatr Scand.

[193] Zanarini MC, Temes CM, Frankenburg FR, Reich DB, Fitzmaurice GM (2020). Levels of acceptance and forgiveness reported by patients with BPD and personality-disordered comparison subjects over 20 years of prospective follow-up. J Personal Disord.

[194] Zanarini MC, Temes CM, Magni LR, Aguirre BA, Hein KE, Goodman M. Risk factors for borderline personality disorder in adolescents. J Personal Disord. 2019:1–8. 10.1521/pedi_2019_33_425. 10.1521/pedi_2019_33_425 PMC7584111 30920936

[195] Ziobrowski HN, Buka SL, Austin SB, Sullivan AJ, Horton NJ, Simone M, Field AE (2020). Using latent class analysis to empirically classify maltreatment according to the developmental timing, duration, and co-occurrence of abuse types. Child Abuse Negl.

